# Developmental-stage-dependent transcriptional response to leukaemic oncogene expression

**DOI:** 10.1038/ncomms8203

**Published:** 2015-05-28

**Authors:** Kakkad Regha, Salam A. Assi, Olga Tsoulaki, Jane Gilmour, Georges Lacaud, Constanze Bonifer

**Affiliations:** 1 School of Cancer Sciences, Institute for Biomedical Research, University of Birmingham at Edgbaston, Birmingham B15 2TT, UK; 2 CRUK Manchester Institute, The University of Manchester, Manchester M20 4BX, UK

## Abstract

Acute myeloid leukaemia (AML) is characterized by a block in myeloid differentiation the stage of which is dependent on the nature of the transforming oncogene and the developmental stage of the oncogenic hit. This is also true for the t(8;21) translocation that gives rise to the RUNX1-ETO fusion protein and initiates the most common form of human AML. Here we study the differentiation of mouse embryonic stem cells expressing an inducible RUNX1-ETO gene into blood cells as a model, combined with genome-wide analyses of transcription factor binding and gene expression. RUNX1-ETO interferes with both the activating and repressive function of its normal counterpart, RUNX1, at early and late stages of blood cell development. However, the response of the transcriptional network to RUNX1-ETO expression is developmental stage specific, highlighting the molecular mechanisms determining specific target cell expansion after an oncogenic hit.

## Abstract (web summary)

Acute myeloid leukaemia often originates from a chromosomal translocation creating a RUNX1/ETO fusion protein. Here Regha *et al*, generate a mouse stem cell model and demonstrate the fusion protein disrupts transcription in a differentiation-stage-specific manner.

## Graphical abstract

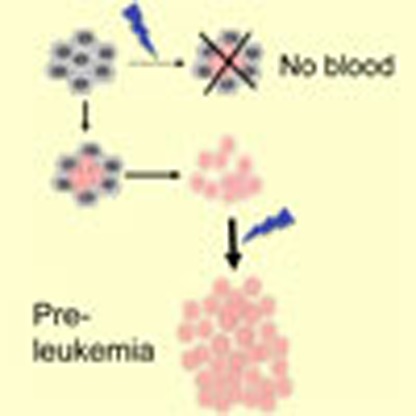

Normal blood cell development originates from haematopoietic stem cells, which can both self-renew and differentiate, and proceeds via the formation of transiently amplifying progenitor cells, which become progressively restricted in their differentiation potential until they arrive at the terminally differentiated state. These cell fate changes are tightly controlled by the interplay between transcription factors (TFs) and the epigenetic machinery and lead to differential gene expression. In addition, cell proliferation in progenitors has to be tightly controlled. Normal blood cell development can be blocked in a number of ways. The most important mechanisms involve (i) the mutation of TFs or epigenetic regulators, (ii) altered functions of such regulators by fusing them to other proteins by chromosomal translocations and (iii) aberrant signalling processes impacting on the activity of both TFs and epigenetic regulatory proteins^1^. Such mutations interfere with the highly coordinated changes in gene expression during haematopoiesis and are the main cause for human leukaemia. Acute myeloid leukaemia (AML) affects the myeloid lineage of the haematopoietic system, which gives rise to granulocytes and macrophages. In this disease, haematopoietic differentiation is blocked at the progenitor stage, giving rise to rapidly proliferating leukaemic blast cells. Depending on the molecular cause of their transformation, leukaemic blast cells are blocked at different (early or late) stages along the myeloid differentiation pathway, indicating (i) that the nature of the oncogenic hit determines the molecular outcome of the transformation event and (ii) that the transcriptional network within a specific target cell is reprogrammed to adopt an alternative differentiation state, which has to be compatible with self-renewal. Currently, the molecular details of how this occurs is unclear.

Studies of leukaemic oncogenes have been instrumental with respect to identifying regulators of normal haematopoiesis^2^. This is exemplified by the gene encoding the TF RUNX1, which is a frequent target of leukaemic mutations. It is also absolutely required for the specification of haematopoietic stem cells in the embryo, but once these are formed, the immediate effect of the knockout is much milder^3^,^4^. The t(8;21) translocation that gives rise to the fusion protein RUNX1-ETO blocks differentiation at an early myeloid progenitor stage^5^ by binding to a subset of RUNX1-target regions^6^. RUNX1-ETO expression is mostly associated with gene repression^7^ and fusion transcripts can be detected in *utero*, indicating that this genetic change can occur early during embryogenesis^8^. However, similar to the RUNX1 knockout, the developmental stage at which the oncogenic hit occurs is of the essence for the resulting phenotype^9^. Experiments in mice showed that germline expression of RUNX1-ETO disrupts embryonic haematopoietic and endothelial development with a complete absence of foetal liver haematopoiesis. In contrast, conditional expression in myeloid progenitors after birth creates cells with enhanced self-renewal capacity that, after the acquisition of additional mutations, become fully malignant^10^,^11^,^12^,^13^. These experiments indicate that the developmental stage at which RUNX1-ETO is expressed determines whether AML develops or not. It was suggested that RUNX1-ETO interferes with RUNX1 function in the embryo, however, no system-wide studies have so far been undertaken to confirm this idea. It is unknown whether RUNX1-ETO targets different cis-regulatory elements during haematopoietic specification in the embryo and in myeloid progenitor cells after birth and how it interferes with RUNX1 function. Here we addressed these questions and describe a global analysis of RUNX1-ETO action before and after the formation of haematopoietic cells, using an inducible version of RUNX1-ETO and embryonic stem (ES) cell differentiation as a model, as this differentiation system faithfully recapitulates all stages of embryonic haematopoietic specification and produces myeloid precursor cells capable of terminal differentiation into macrophages. We show that RUNX1-ETO interferes with both the repressive and the activating action of RUNX1 and targets a similar set of genes at both stages but causes a different gene expression response. Our data demonstrate that not the target genes but the response of the transcriptional network within target cells is the main determinant of the outcome of an oncogenic hit.

## Results

### Developmental-stage-specific outcome of RUNX1-ETO induction

**Figure 1:**
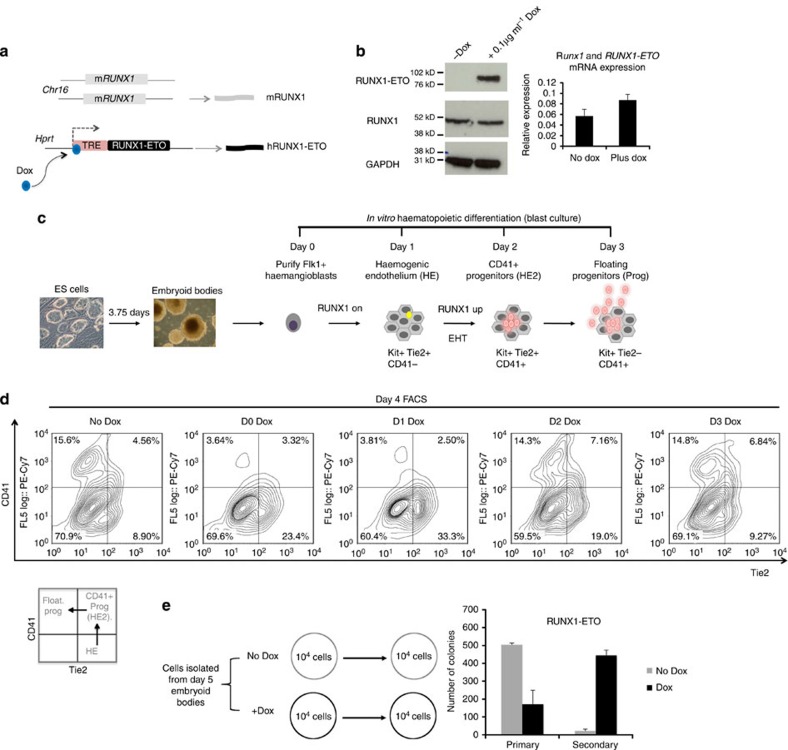
Induction of RUNX1-ETO at early and late stages of haematopoietic specification has a different outcome. (**a**) Schematic representation of the RUNX1-ETO inducible ES cell line. (**b**) RUNX1-ETO expression levels are physiological. Western blot measuring RUNX1-ETO, RUNX1 and GAPDH (loading control) protein expression in total lysates from uninduced myeloid progenitor cells or cultures induced overnight with 0.1 μg ml^−1^ doxycycline as indicated. All samples are from the same gel and all signals were obtained using the same exposure time. The original blot can be found in Supplementary Fig. 8. Right panel: analysis of expression of *Runx1* and *RUNX1-ETO* mRNA from uninduced and induced myeloid precursor cells using primers against a highly conserved part of the RUNT domain encoding sequence demonstrating that the increase in *RUNX1-ETO* expression does not exceed that of *Runx1*. Error bars represent the s.d. of three independent experiments. (**c**) Schematic diagram representing the outline of *in vitro* haematopoietic differentiation and the development of the different types of cells. (**d**) Developmental-stage-dependent impact of RUNX1-ETO. RUNX1-ETO was induced at the indicated days of blast culture and developing cells from induced and control cultures were analysed by flow cytometry at day 4 after staining with antibodies against Tie2, CD41 and KIT. (**e**) Cells derived from day 5 EBs were serially replated in colony-forming unit assays in presence or not of doxycyline. Average numbers (and standard errors) of definitive haematopoietic colonies generated by 10^4^ cells replated in triplicates are depicted.

To be able to examine the effect of RUNX1-ETO expression at different stages of blood cell development, we constructed an ES cell line carrying a doxycycline (Dox)-inducible human RUNX1-ETO (*RUNX1T1)* gene that is expressed from a tetracycline (TET)-responsive promoter in a RUNX1 wild-type genetic background (Fig. 1a; Supplementary Fig. 1a). The system is tightly regulated as no RUNX1-ETO protein is detected in the absence of Dox (Fig. 1b). It has recently been shown in t(8;21) AML that a balance between RUNX1 and RUNX1-ETO expression is required for maintaining the leukaemic phenotype^14^. We, therefore, carefully titrated the Dox concentration and found that 0.1 μg ml^−1^ was the optimal concentration for the levels of RUNX1-ETO expression not exceeding that of expression of the endogenous *Runx1* protein and messenger RNA (mRNA; Fig. 1b). ES cells were then differentiated into haematopoietic cells using a previously described culture system (blast culture) based on seeding Flk1^+^ cells containing common precursors for haematopoietic and endothelial cells, that is, haemangioblasts^15^ (Fig. 1c). Haematopoietic specification from the haemangioblast stage progresses via an adherent haemogenic endothelium (HE) cell type expressing the endothelial marker Tie2 and starting to express the receptor for the stem cell factor KIT on the surface. The HE expresses a low level of RUNX1, which is required to induce the endothelial program but is not sufficient to initiate the formation of haematopoietic cells^16^. Haematopoietic development is initiated by the upregulation of RUNX1, which drives the endothelial–haematopoietic transition (EHT) during which cells acquire the CD41 surface marker (hereby referred to as HE2) and are fully committed to form blood^9^,^15^,^17^. The loss of the endothelial marker Tie2 marks the stage when cells round up and become floating multipotent progenitors expressing both the surface markers KIT and CD41.

We induced RUNX1-ETO at different time points during blast culture (day 0–3) and analysed the surface marker expression of cells by flow cytometry (Fig. 1d; Supplementary Fig.1b). To this end, we stained cells with antibodies against KIT, Tie2 and CD41 whose individual or co-expression marks mesoderm-derived cells on their way to becoming haematopoietic progenitors^15^,^18^,^19^ as described above and depicted in Fig. 1c. Figure 1d shows that the time point of RUNX1-ETO induction determined the outcome of differentiation. When cells were induced at day 0 and day 1, KIT expression was not affected (Supplementary Fig. 1c), CD41 was not upregulated and differentiation did not proceed further. Induction at day 0 of the expression of RUNX1-ETO9a, a splice variant of RUNX1-ETO which gives rise to a truncated protein that is capable of causing leukaemia in mice on its own^20^, resulted similarly in a defect in generation of CD41+ cells (Supplementary Fig. 2a,b).

When expression of RUNX1-ETO was induced after day 2, KIT^+^CD41^+^Tie2^−^ progenitor cells were formed. When placed in myeloid expansion medium containing cytokines together with Dox, these cells show rapid growth (Supplementary Fig. 1d,e). Moreover, RUNX1-ETO-expressing progenitor cells displayed an enhanced self-renewal capacity as shown by serial replating assays (Fig. 1e; Supplementary Figs 1f and 2c). Taken together, these *in vitro* experiments confirm the results of the earlier mouse studies. However, they add the information that the block in differentiation at early stages of embryonic haematopoietic specification occurs precisely at the EHT.

### RUNX1-ETO induction differentially alters gene expression

**Figure 2:**
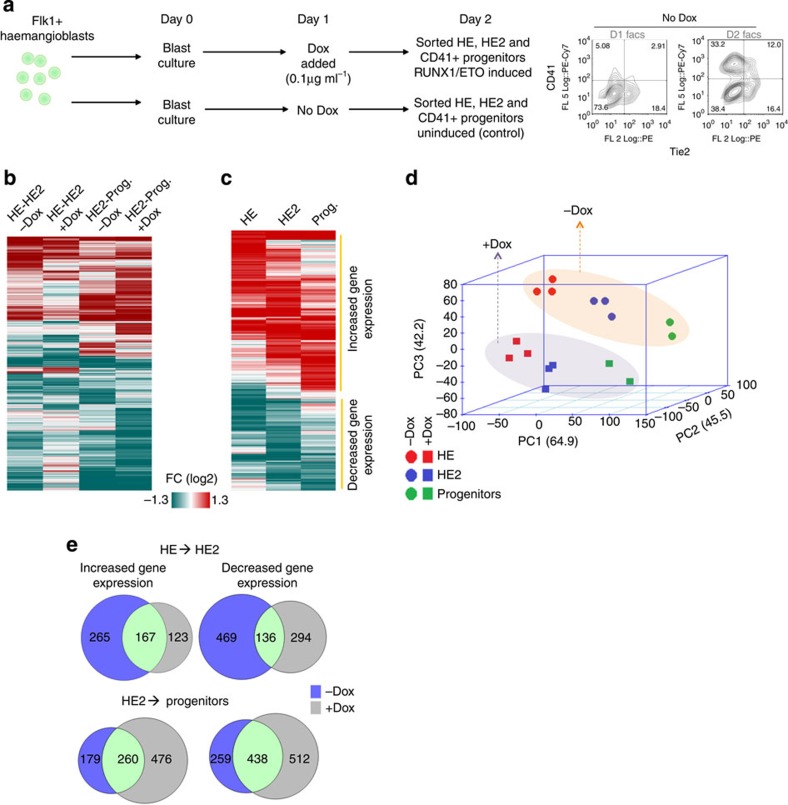
RUNX1-ETO induction alters the expression of different genes depending on the developmental stage. (**a**) Schematic diagram of the FACS purification of HE, HE2 and CD41^+^ progenitors for microarray analysis. Right panel: surface marker profile of uninduced cultures at the indicated days. (**b**) Hierarchical clustering of the fold change of the differentially expressed genes through different stages of differentiation from HE to HE2 and HE2 to progenitors before and after day 1 Dox induction. (**c**) Heat map showing hierarchical clustering of the fold change of 2,718 differentially expressed genes between the induced and the uninduced state after RUNX1-ETO induction. (**d**) Principal component analysis of gene expression of uninduced and induced samples indicating that RUNX1-ETO differentially alters the expression profiles of genes at each stage. (**e**) Venn diagrams representing overlapping or distinct up- or downregulated genes through differentiation, between HE and HE2 (top) and between HE2 and progenitors (bottom).

The expression of RUNX1-ETO changes the gene expression pattern of haematopoietic precursor cells, both in human and in mouse models^6^,^21^,^22^,^23^. We next examined which genes were deregulated at the different developmental stages when RUNX1-ETO was induced. To examine the gene expression changes responsible for the block of the EHT, we induced RUNX1-ETO at day 1 of blast culture (Fig. 2a; Supplementary Data 1) at which RUNX1 expression levels are very low^24^. However, these developing cell populations are not completely synchronous and contain some cells already having progressed further in differentiation at the point of induction. We, therefore, purified induced and non-induced cells according to their surface marker expression by cell sorting for measurement of global gene expression using microarrays. The isolated cells represented the HE, HE2 (CD41^+^ cells still expressing Tie2) and progenitor cell (Tie2-negative CD41^+^KIT^+^) populations (Supplementary Fig. 2d). These experiments demonstrated that the normal course of gene expression changes during differentiation was disrupted by induction of RUNX1-ETO (Fig. 2b,c; Supplementary Fig. 2f). Moreover, the comparison of gene expression patterns by principal component analysis demonstrated that HE2 cells expressing RUNX1-ETO displayed a gene expression profile that was closer to that of the earlier HE stage (Fig. 2d; Supplementary Fig. 2e). This was also true for CD41^+^ KIT^+^Tie2^−^ progenitor cells. Each population showed a different shift in the gene expression pattern depending on which stage RUNX1-ETO expression had taken effect. Both normally upregulated and downregulated genes belonging to different gene ontology classes (Supplementary Data 2) were affected (Fig. 2c,e), displaying complex deregulation patterns (Supplementary Fig. 3a).

**Figure 3:**
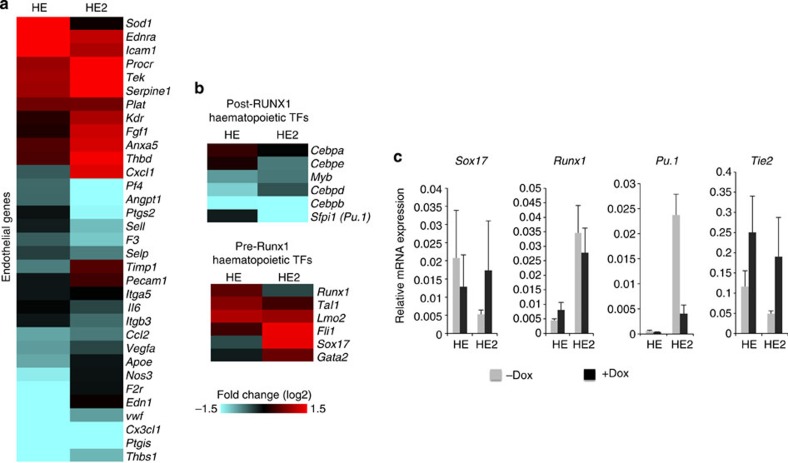
RUNX1-ETO induction disturbs differentiation-dependent gene expression changes during the EHT. (**a**) Heat map representing the fold change of endothelial gene expression between RUNX1-ETO-induced and uninduced cultures in the HE and HE2. Each line represents the RNA fold change in expression of individual genes between the induced and uninduced cultures. Red and blue indicate increased and decreased expression, respectively. (**b**) RUNX1-ETO expression regulates pre- and post-RUNX1 transcription factors genes differently. Heat map representing changes in expression of haematopoietic transcription factors between induced and uninduced cultures in the HE and HE2. Each line represents the RNA fold change in expression of individual genes between the induced and uninduced cultures. (**c**) PCR with reverse transcription validation of microarray results using RNA isolated from HE and HE2 of the RUNX1-ETO-induced and uninduced cultures. Error bars represent the standard error of three independent experiments.

We examined two classes of genes in more detail. After the EHT, a number of important haematopoietic regulator genes are upregulated in HE2 in response to the increase in RUNX1 levels. At the same time, also due to RUNX1 action, endothelial genes are first up- and then downregulated thus firmly committing cells to the blood cell lineage^16^,^25^. This process is strongly disturbed after RUNX1-ETO induction as only very few Tie2^−^ floating progenitor cells were formed, indicating that the EHT was perturbed (Fig. 1d). Multiple endothelial genes and TF-encoding genes characteristic for the HE such as *Sox17* remained expressed and the expression of others is deregulated (Fig. 3a; Supplementary Fig. 3b), while a number of TF genes important for myelopoiesis, such as *Sfpi1(Pu.1)* are not upregulated (Fig. 3b,c; Supplementary Fig. 3c,d). In essence, the entire transcriptional network of these developing cells is reprogrammed as multiple TF genes are being deregulated (Supplementary Fig. 3c,d)

**Figure 4:**
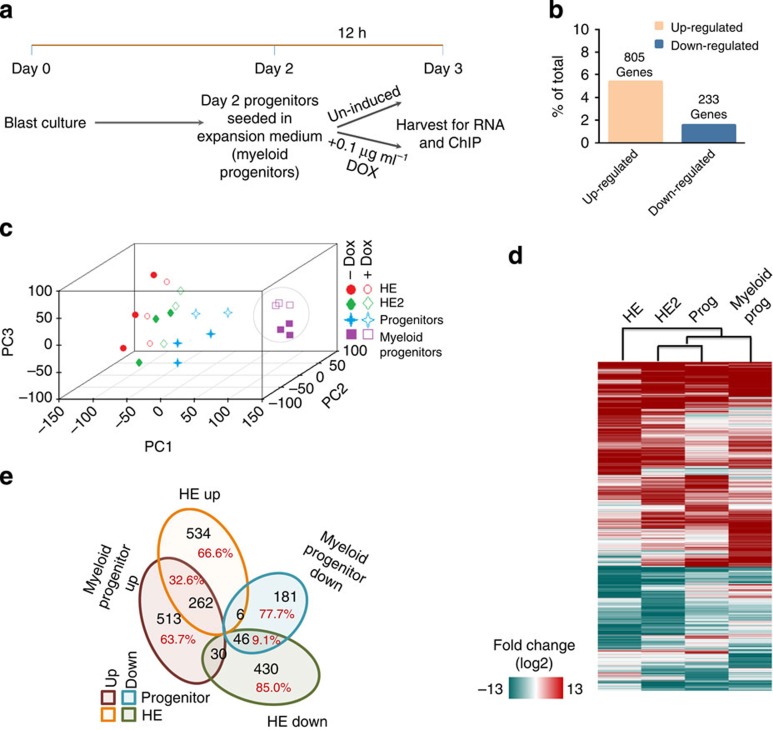
Induction of RUNX1-ETO in committed myeloid progenitor cells. (**a**) Outline of myeloid progenitor culture and induction strategy in progenitor expansion medium. (**b**) Percentage and number of up- and downregulated genes in myeloid progenitor cultures after overnight RUNX1-ETO induction compared with all expressed genes. The numbers over the bars represent the number of genes changing expression. (**c**) Principal component analysis demonstrating the differential effect of RUNX1-ETO induction on gene expression in four different cell types induced at different stages (HE, HE2, CD41^+^KIT^+^Tie2^−^ progenitors and myeloid progenitors). RUNX1-ETO-induced gene expression changes in myeloid progenitors is markedly different from that in early progenitors where RUNX1-ETO was induced in the HE stage. (**d**) Heat map demonstrating differential gene expression change of direct RUNX1-ETO targets in the different cell types after RUNX1-ETO induction. (**e**) Venn diagram demonstrating that there is little overlap between RUNX1-ETO-target genes changing expression in the different cell types.

To compare gene expression changes induced by RUNX1-ETO between early stages of haematopoietic specification and blood progenitors, we prepared RNA from purified KIT^+^CD41^+^ multipotent progenitor cells from the blast culture, seeded them in expansion medium supplemented with cytokines to generate committed myeloid progenitor cells (myeloid progenitors) and at the same time induced RUNX1-ETO for 12 h (Fig. 4a; Supplementary Fig. 4a). We also prepared chromatin for chromatin immunoprecipitation (ChIP) assays (Fig. 4a). Expression array analysis from these cells shows 805 upregulated and 233 downregulated genes (Fig. 4b) displaying a gene expression pattern that is distinct to that of the earlier CD41^+^KIT^+^Tie2^−^ multipotent progenitors (Fig. 4c; Supplementary Fig.4b). We recently measured the transcriptional response of short interfering RNA-mediated RUNX1-ETO depletion in human t(8;21) cells^6^. The comparison of these changes using Gene Set Enrichment Analysis (GSEA) demonstrates that there is a strong inverse correlation between the gene expression changes after induction of RUNX1-ETO and after depletion (Supplementary Fig. 4c). However, the pattern of gene expression changes after RUNX1-ETO induction in committed myeloid progenitors differs from that observed at earlier stages (Fig. 4d,e; Supplementary Fig. 4c,d) and at the myeloid progenitor stage (Supplementary Fig. 4e). Only 9.1% of the genes downregulated in the HE and 32.6% of those upregulated also change expression in myeloid progenitors (Fig. 4e). This result indicates that the transcriptional networks of the two cell types respond differently to RUNX1-ETO induction.

### RUNX1-ETO binds to a core set of HE and progenitor genes

**Figure 5:**
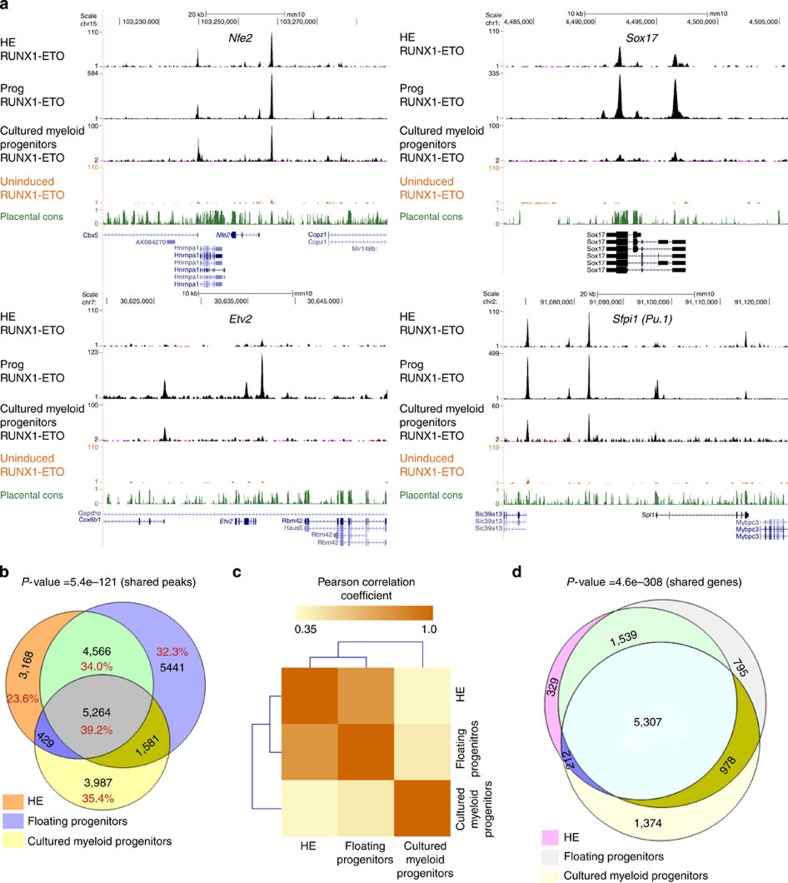
RUNX1-ETO binds to different binding sites but similar genes at early and late stages. (**a**) UCSC genome browser screenshots of ChIP-seq data for RUNX1-ETO in HE, CD41^+^KIT^+^Tie2^−^ progenitors and cultured myeloid progenitors at *Nfe2, Sox17, Etv2* and *Sfpi1 (Pu.1)*, (**b**) Venn diagram showing the percentage and number of intersections between the RUNX1-ETO ChIP-seq peaks in HE, progenitors and myeloid progenitors. The *P* value for calculating the significance of the overlap of all shared peaks (5,264) was obtained by bootstrapping and it was found to be highly significant, with z scores of 23.36 (*P* value<5.45e−121). (**c**) Pearson correlation analysis of the ChIP-seq data obtained with HE, progenitors and myeloid progenitors demonstrating that HE and progenitors from the blast culture cluster away from the myeloid progenitors, which have progressed further in differentiation. Pearson’s correlation coefficients were calculated between all union peaks using the normalized read counts. A correlation matrix was generated and Pearson correlation coefficients are displayed after hierarchical clustering as a heat map. (**d**) Venn diagram showing the overlap of RUNX1-ETO bound genes in HE, progenitors and cultured progenitors. The shared gene overlap was found to be significant with *P* value<4.6e−30 (ref. 8).

One reason for the differential response of haemogenic endothelial cells and myeloid progenitor cells could be that RUNX1-ETO binds to different target sites. To this end, we determined the binding sites for RUNX1-ETO by ChIP using an antibody against its HA-tag at three different stages: HE, CD41^+^KIT^+^Tie2^−^progenitors induced at day 2 (Supplementary Fig. 5a,b) and from the myeloid progenitors grown in expansion medium, each of which was induced for 12 h. Analysis of the ChIP data shows that RUNX1-ETO binds to several thousand sites in each cell type (Supplementary Fig. 5c, with examples shown in Fig. 5a). The comparison between the binding sites (Fig. 5b,c) reveals that there is a significant similarity (73% overlap) between the binding patterns of HE and CD41^+^KIT^+^Tie2^−^progenitors, whereas only 42% of HE peaks coincide with those from cultured myeloid progenitors, reflecting the differences in gene expression profiles (Fig. 4c,d,e). However, there is a core of >5,000 peaks that is shared between all data sets. Interestingly, when we assign binding sites to specific genes by matching them to the nearest promoter, the overlap at the gene level is significantly higher (Fig. 5d). This result indicates that RUNX1-ETO targets similar genomic neighbourhoods in all three analysed cell types.

In human t(8;21) cells, RUNX1-ETO forms a complex with E-Twenty-Six (ETS) factors (FLI-1 and ERG) and E-Box-binding proteins (SCL/TAL1, LYL and HEB)^7^,^26^,^27^. To examine whether this was also the case in the HE and in CD41^+^KIT^+^Tie2^−^progenitors, we conducted an unbiased search for enriched DNA sequence motifs at distal RUNX1-ETO-binding sites shared between HE and CD41^+^KIT^+^Tie2^−^progenitors (9,830 peaks; Fig. 5b) and all three populations (5,264 peaks; Fig. 5b) and confirmed that here RUNX1-ETO peaks are also enriched for binding motifs for these three factors (Supplementary Fig. 5d).

**Figure 6:**
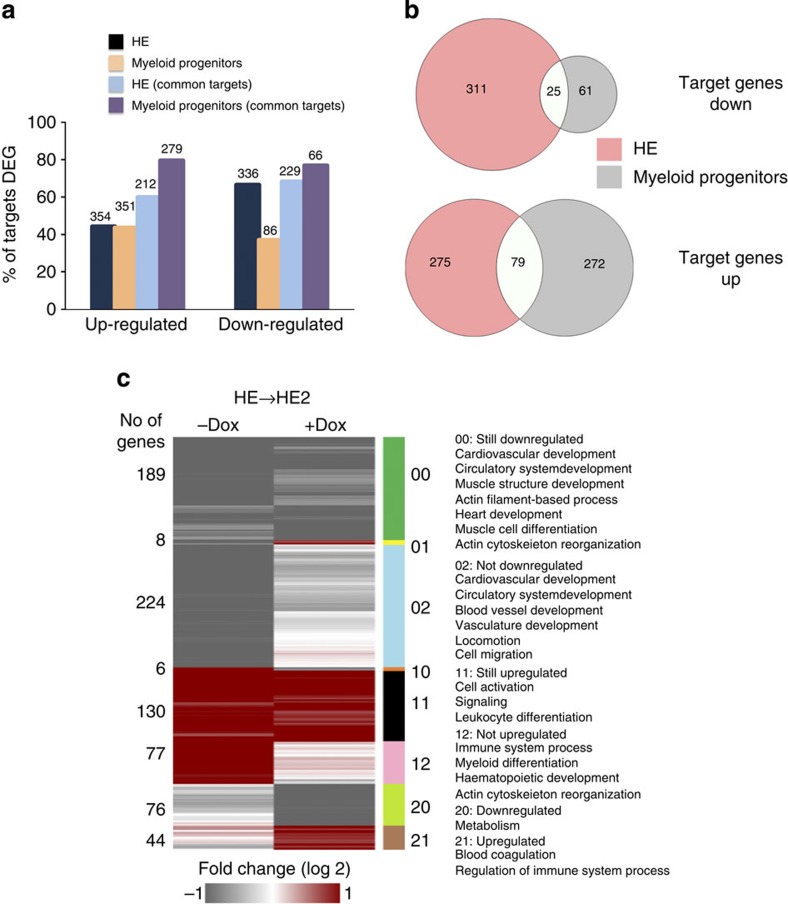
The response of the transcriptional network to RUNX1-ETO induction is stage specific. (**a**) Graph depicting the number and proportion of all and shared (common) RUNX1-ETO targets among differentially expressed genes (DEGs) in the different cell populations. (**b**) Venn diagram showing only a small overlap between the downregulated (top) and upregulated (bottom) transcripts from RUNX1-ETO-target genes in the HE and in cultured myeloid progenitors after RUNX1-ETO induction. (**c**) Analysis of the influence of RUNX1-ETO binding on the kinetics of upregulation and downregulation of genes during the EHT. The heat map shows an unbiased clustering of the fold change in gene expression during the EHT with and without RUNX1-ETO induction with the different clusters (00, 01, 02 and so on) indicated on the right. Right panel: predominant gene ontology terms of genes belonging to the different clusters.

Changes in gene expression can be caused by direct RUNX1-ETO binding or indirectly. We, therefore, integrated the ChIP-sequencing (ChIP-seq) data from HE and myeloid progenitors with the global expression data from both populations. This analysis demonstrates that (i) about 50% of RUNX1-ETO-responsive genes are direct targets and (ii) that expression of only a minority of genes bound by RUNX1-ETO is influenced by RUNX1-ETO binding (Fig. 6a). However, similar to the global comparison of gene expression (Fig. 4c,d,e), and in spite of the strong similarity of RUNX1-ETO-associated genes (Fig. 5a,d) there is a striking difference in the response of RUNX1-ETO-target genes to RUNX1-ETO induction (Fig. 6b). This difference is also reflected in the pathways affected by RUNX1-ETO induction (Supplementary Fig. 6a–d). RUNX1-ETO-target genes upregulated in the HE (Fig. 6a) are involved in focal and cell adhesion, metabolism and MAP kinase (MAPK) signalling, indicative of the block of the EHT; downregulated genes (Fig. 6b) affect various other signalling pathways such as chemokine and cytokine genes (Supplementary Data 3). In progenitors, RUNX1-ETO induction upregulates a large number of TF genes characteristic for the stem cell stage, such as *Erg*, *Fli1* and *Meis1*, but also genes encoding for signalling molecules such as the Notch ligand *Jag1*, which plays a role in early haematopoietic specification^28^ (Supplementary Fig. 6c). Downregulated-target genes involve numerous genes involved in cell signalling as well as the gene encoding the myeloid master regulator PU.1 (*Sfpi1*) and members of the C/EBP family of TFs such as *Cebpb/Cebpa* all of which are important for myelopoiesis^29^,^30^,^31^,^32^ (Supplementary Fig. 6c), explaining enhanced cell renewal in progenitor cells and the block in myeloid differentiation. Recently, a mouse model of t(8;21) was developed that recapitulated the different steps in leukaemogenesis from the pre-leukaemic to the leukaemic stage^33^. The comparison of the gene expression profiles of our ES cell-derived cells and primary murine early pre-leukaemic progenitors shows a high level of concordance of gene expression changes in RUNX1-ETO-expressing cells (Supplementary Fig. 6e), thus validating our *in vitro* differentiation model.

RUNX1-ETO binding is mostly associated with gene repression^7^. We, therefore, analysed how the binding of RUNX1-ETO to its targets affects the kinetics of upregulation and downregulation of genes during the EHT. To this end, we clustered RUNX1-ETO targets according to their expression behaviour with and without induction (Fig. 6c). This analysis reveals that RUNX1-ETO binding affects many, but not all differentially expressed target genes in the same way, indicating that the impact of its expression is not solely repressive. While a number of genes do not change their expression kinetics including a large group of genes involved in heart and muscle development, several groups of genes are not upregulated or not downregulated. The former (77 genes, group 12) contains genes associated with haematopoietic differentiation, and the latter (group 02) contains 224 genes that associate with blood vessel and vasculature development, thus identifying the core network of HE genes affected by RUNX1-ETO binding.

In summary, these data show that the global transcriptional response to RUNX1-ETO induction is dictated not by the nature of its target genes, but by the target cell type and its transcriptional network.

### RUNX1-ETO interferes with RUNX1 binding

**Figure 7:**
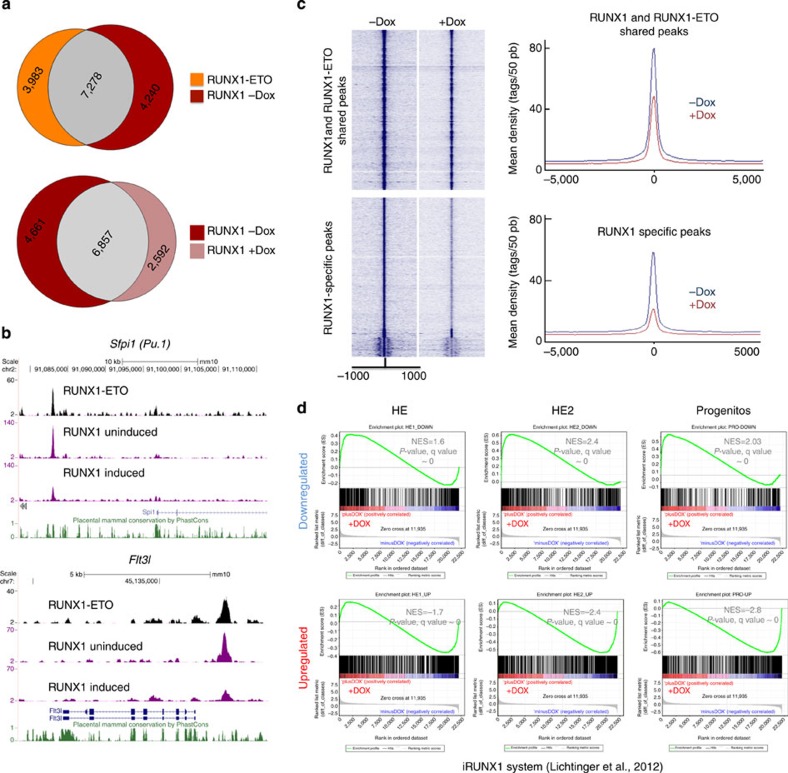
RUNX1-ETO causes the disruption of pre-existing RUNX1 complexes and interferes with the activating and repressive function of RUNX1. (**a**) Venn diagram showing the overlap of RUNX1-ETO and RUNX1 ChIP-seq peaks in the cultured progenitors after induction (top). The bottom diagram shows the overlap of the RUNX1 peaks in the uninduced and the induced cultured progenitors. (**b**) Screenshots showing a reduction in RUNX1 ChIP-seq peaks across the indicated loci. (**c**) Composite RUNX1 ChIP-seq peak distribution profiles within 1 kb of the peak centre. The top diagram shows the relative tag densities for the peaks shared between the uninduced and the induced progenitors in blue and brown, respectively. The bottom diagram shows the reduction in tag densities of the RUNX1-specific peaks in the uninduced cultures. (**d**) GSEAs correlating the gene expression profiles at the indicated stages with or without RUNX1-ETO obtained in this study with those from RUNX1 knockout HE with or without induction of RUNX1 (ref. 17), demonstrating an inverse correlation of responses.

In the absence of RUNX1, cells are blocked at the HE stage, but most factors specifying haematopoietic cells such as SCL/TAL1 and FLI1 are already expressed and bind to their target genes^17^. Moreover, although expressed at a low level in the HE, RUNX1 already interacts with specific targets^16^,^24^. High levels of RUNX1 are required to induce myeloid differentiation genes such as *Cebpa, Sfpi1* and *Irf8*. This suggests that the crucial differences in the transcriptional networks of HE and progenitor cells are caused by differential levels of RUNX1 and that RUNX1-ETO could directly interfere with RUNX1 binding to its targets. Using an short interfering RNA approach in human t(8;21) cells, we showed that RUNX1-ETO binds to a subset of RUNX1 sites and is replaced by RUNX1 once it has been depleted^7^. To test whether RUNX1-ETO can displace pre-existing RUNX1 complexes, we determined RUNX1 binding in cultured myeloid progenitor cells before and after RUNX1-ETO induction. As in human cells, RUNX1-ETO and RUNX1 share at least 65% of their binding sites (Fig. 7a, with examples shown in Fig. 7b) but RUNX1 also binds to specific sequences on its own. The induction of RUNX1-ETO causes a general reduction in RUNX1 binding (Fig. 7b,c; Supplementary Fig. 7a; with examples shown in Fig. 7b) without a concomitant reduction in the RUNX1 protein levels (Fig. 1b). RUNX1-ETO is in direct competition with RUNX1 at shared binding sites as RUNX1 binding is reduced after RUNX1-ETO induction (Fig. 7a,b,c; Supplementary Fig. 7b). In human progenitor cells, RUNX1 associates with other factors, predominantly C/EBPα^7^. To test why RUNX1-only sites are also lost after induction, we determined the motif composition and density of other motifs within RUNX1 ChIP peaks. We found a significant enrichment of ETS and GATA motifs but now also C/EBP motifs (Supplementary Fig.7c). In RUNX1-only sites, C/EBP motifs co-localize with RUNX motifs within the peaks (Supplementary Fig.7d). Sites bound by both RUNX1-ETO and RUNX1 co-localize with ETS and to a lesser extent with C/EBP motifs (Supplementary Fig.7e). Co-localization of RUNX, ETS and C/EBP but not GATA motifs in RUNX1-only peaks is significant as shown by bootstrapping analysis (Supplementary Fig.7f), suggesting that the reduction of RUNX1 binding was associated with the downregulation of C/EBP and ETS (most likely PU.1) proteins.

We recently used an ES cell differentiation system carrying an inducible version of RUNX1 in a *RUNX1*^−/−^ genetic background to show that the induction of RUNX1 leads to a shift in TF binding towards the new RUNX1-binding sites^17^. It is well established that besides its role as transcriptional activator, RUNX1 can also act as a transcriptional repressor^34^. Figure7d demonstrates a strong inverse correlation between the gene expression changes caused by the induction of RUNX1-ETO and those induced by RUNX1 in the HE. This was true for both upregulated and downregulated genes.

Taken together, our data demonstrate that RUNX1-ETO directly competes with RUNX1 binding at a subset of target sites both in the HE and in progenitor cells. In addition, RUNX1-ETO induction alters the transcriptional network of myeloid progenitors, and thus affects the cooperative binding of RUNX1 with other factors. It, thus, interferes with both aspects of RUNX1 function, gene activation and gene repression.

## Discussion

**Figure 8:**
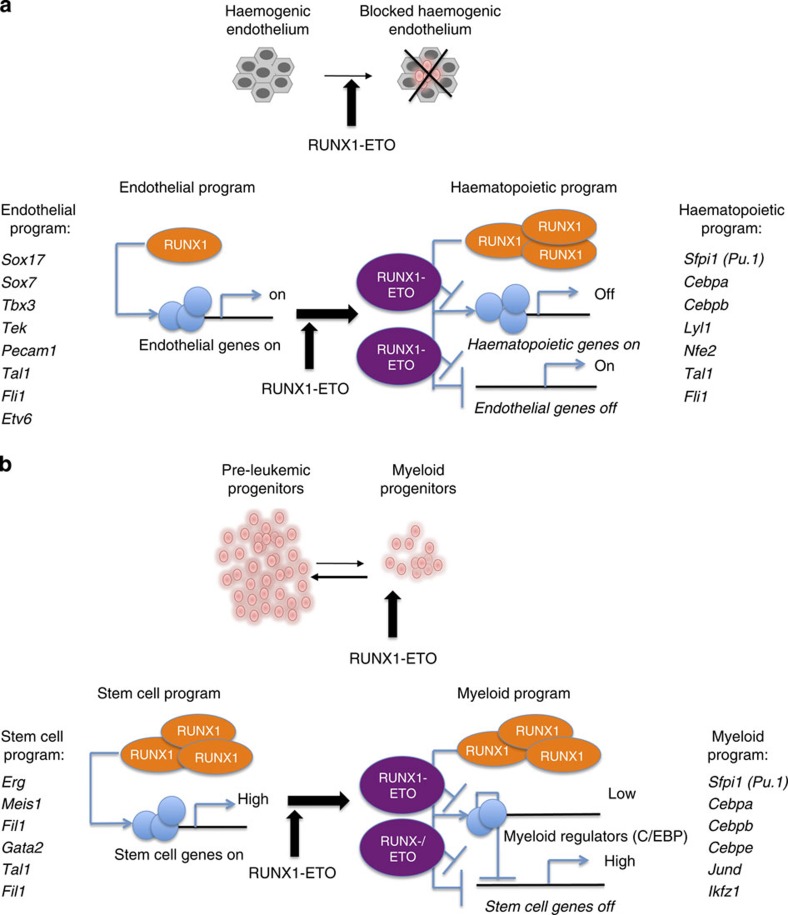
Model of RUNX1-ETO-mediated gene expression changes in two different target cell types. (**a**) Expression of RUNX1-ETO in the HE leads to a block in the EHT. Left panel: endothelial gene expression program with low levels of RUNX1 and expression of endothelial genes. Right panel: haematopoietic gene expression program where haematopoietic genes are upregulated and endothelial genes are downregulated. The induction of RUNX1-ETO leads to a lack of upregulation of haematopoietic genes (indicated as ‘off’) but also a failure to repress the endothelial genes (indicated as ‘on’). (**b**) Expression of RUNX1-ETO in myeloid progenitors leads to enhanced self-renewal. Left panel: stem cell gene expression program including early haematopoietic regulator genes, this is normally only transiently observed in ES-derived cells (see Fig.3 and Supplementary Fig. 3). Right panel: myeloid gene expression program found in committed myeloid progenitor cells. Here the stem cell program is downregulated, while the myeloid program is upregulated. RUNX1-ETO interferes with this downregulation by targeting RUNX1 sites at myeloid regulator genes (indicated as ‘low’), which then in turn fail to downregulate the stem cell program (indicated as ‘high’).

In this study, we addressed at the systems level the question of how a single oncogenic TF can exert different effects depending on the cell type in which it is expressed (Fig. 8). After induction in the HE and in progenitor cells, RUNX1-ETO binds to a similar set of genes. When expressed in the HE (Fig. 8a), RUNX1-ETO interferes with the effects of RUNX1 upregulation and the EHT is impaired as cells are unable to upregulate haematopoietic differentiation genes and downregulate the endothelial gene expression program. However, deregulation goes beyond the direct target genes. Multiple TF genes and genes regulating focal adhesion are deregulated, which is likely to account for the inability of these cells to undergo the morphological changes occurring through the EHT. In haematopoietic progenitors (Fig. 8b), RUNX1-ETO shifts cells to a more stem cell-like state and delays myeloid differentiation by rapidly downregulating myeloid differentiation genes with a concomitant upregulation of normally downregulated stem cell genes, such as *Erg*. The latter phenomenon lends weight to the idea that the upregulation of myeloid regulators such as C/EBPα could negatively feed back to stem cell genes as demonstrated for *SOX4* (ref. 35). In addition, induced cells upregulate multiple genes encoding signal transduction molecules, including multiple MAPK genes and show signs of chronic signalling by displaying an increase in expression of the MAPK inducible TF genes *Fos, Fosb* and *JunD*. RUNX1-ETO cannot cause leukaemia on its own but requires secondary mutations, mostly in growth factor receptor genes^13^,^36^,^37^. Our data demonstrate that RUNX1-ETO already activates growth-stimulating signals. Together with the downregulation of differentiation driving TFs, this may account for the partial block in differentiation and enhanced self-renewal.

We show that RUNX1-ETO directly interferes with RUNX1 function at both developmental stages and can disrupt previously existing RUNX1 complexes. However, while RUNX1-ETO acts as a repressor, its effect on gene expression within the transcriptional network is both positive and negative. At each responsive gene, the disruption of RUNX1-containing complexes occurs by both direct and indirect mechanisms involving other TF families. In the HE, RUNX1-ETO induction is likely to interfere with RUNX1 binding as upregulation of direct RUNX1-target genes is abolished. In myeloid progenitor cells, RUNX1-ETO induction leads to a reduction of pre-existing RUNX1 complexes at shared binding sites by direct competition but it also reduces binding at RUNX1-only sites. The reason for the latter is most likely the RUNX1-ETO-mediated downregulation of PU.1 and C/EBPα. This idea is supported by our finding that such sites are enriched for motifs for C/EBP family members, but also by our previous studies, which examined the dynamics of TF binding after RUNX1-ETO knockdown^7^. RUNX1-ETO depletion causes the upregulation of C/EBPα, which then cooperates with RUNX1 and PU.1 to bind to thousands of new binding sites and to drive myeloid differentiation. Our data suggest that such complexes fall apart after the RUNX1-ETO-mediated downregulation of myeloid regulators.

The data described here are relevant for leukaemogenesis in general as they shed light on the prerequisites dictating target cell specificity of oncogene action. Our data provide a first insight into why the successful conversion into a pre-leukaemic state occurs at a haematopoietic progenitor stage where cells are capable of activating a self-renewal program and not at the endothelial stage. They also demonstrate that the specific interaction of an aberrant oncogenic TF with the transcriptional network of their target cells is the decisive factor for whether a cell starts on the path to malignancy or not. It will be important to understand the fine details of how oncogenic factors reprogram a core set of target genes and activate self-renewal programs as this will highlight ways of therapeutic intervention.

## Methods

### Construction of the p2lox AE-targeting vector

The HA-tagged RUNX1-ETO and RUNX1/ETO9a fragments from plasmid MigR1 AE (a gift from Christian Wichmann) were cloned into the p2lox-targeting vectors (a gift from Michael Kyba, University of Minnesota).

### Electroporation of ES cells

Approximately 2.5–3.0 × 10^6^ A2lox ES cells (a gift from Michael Kyba) grown on mouse embryonic fibroblasts were harvested by trypsinization, washed with 25 ml PBS (Sigma D8537), resuspended in 125 μl PBS and mixed with 20 μg of each p2lox AE-targeting vector and Cre-expressing plasmid at 240 V, 7 ms settings on a EPI 2500 electroporator (Fischer). Immediately after electroporation, 1 ml ES cell medium with leukaemia inhibitory factor (LIF) was added to the cells, they were plated on feeder cells on a 6-cm Corning dish and were grown for 24 h without selection. ES cell medium was then supplemented with G418 (300 μg per ml) and cells were grown for 5–7 days, changing the medium every day. Individual colonies were picked and expanded on mouse embryonic fibroblasts. The clones were frozen in foetal calf serum with 10% dimethylsulphoxide and stored in liquid nitrogen.

### ES cell culture

ES cells were grown on a layer of feeder cells in DMEM-ES (Sigma D6546) medium supplemented with 15% FCS, 1 mM sodium pyruvate, 1 mM glutamine, 100 units per ml penicillin and 100 μg ml^−1^ streptomycin, 0.15 mM MTG, 25 mM HEPES buffer, 1 × non-essential amino acids (Sigma) and 10^3^ units per ml LIF (ESGRO mLIF, Millipore ESG1107). Medium was changed every day. Before *in vitro* differentiation (IVD), the cells were grown without feeder cells for two passages and the medium used for the last passage was IMDM-ES (Sigma I3390).

### *In vitro* haematopoietic differentiation

ES cells were differentiated into embryoid bodies (EBs)^18^. Briefly, 1 × 10^6^ ES cells previously grown on feeder cells were plated on a gelatinised 10-cm Corning dish in DMEM medium with LIF. The next day the medium was replaced with IMDM-ES and LIF and grown for another day. For *in vitro* differentiation (IVD), cells were harvested and seeded in an *in vitro* differentiation medium in 15-cm Sterilin bacterial plates (Thermo Scientific) at a density of 2.5 × 10^4^ cells per ml. The plates were incubated for 3.75 days during which time cells formed EBs. EBs were harvested and the cells were dispersed by treating the EBs with TrypLE Express (12605–028) and pipetting the cells in and out with a Pasteur pipette (FisherBrand 1346–9108). The cells were passed through a 70-μm cell strainer (Falcon, Cat. No. 352350) and counted. Cells were washed with PBS and resuspended in IMDM with 20% FCS (1 ml per 10^7^ cells). The cells were incubated with biotinylated Flk1 antibody (eBioscience 13-5821-85), 5 μl per 10^7^ cells, and incubated on ice for 15 min. After washing the cells twice with PBS, the cells were incubated with 20 μl of anti-biotin microbeads (Miltenyi Biotec: 130-090-485) per 10^7^ cells for 15 min. The cells were washed with PBS and then loaded onto a MACS column (130-042-401). The column was washed with 9 ml MACS separation buffer (made by diluting MACS BSA stock solution—Miltenyi Biotec 130-091-376—with PBS to a final BSA concentration of 0.5%) and the cells were eluted as per the manufacturer’s instructions. The cells were counted and plated in 15-cm Corning dishes at a density of 10^4^ per ml in blast culture medium (IMDM supplemented with 10% FCS, 100 units per ml penicillin and 100 μg ml^−1^ streptomycin, 1 mM glutamine, 0.45 mM MTG, 0.18 mg ml^−1^ Human transferrin (Roche 10652202001) 25 μg ml^−1^ ascorbic acid, 20% D4T conditioned media, 5 μg l^−1^ recombinant mouse Vascular Endothelial Growth Factor (Peprotech: 450-32), 10 μg l^−1^ mIL-6 (Peprotech: 216–16).

### Flow cytometry analysis of cells from blast cultures

Supernatants of blast culture contain floating progenitor cells. FACS was, therefore, performed on suspension and attached cells. After pelleting the cells, they were stained with a 1:100 dilution of anti-mouse CD202b (TIE2) phycoerythrin (eBioscience: 12-5987-81), 1:50 dilution of allophycocyanin conjugated rat anti-mouse CD117 Clone 2B8 (RUO) (BD Pharmingen: 553356) and 1:100 dilution of anti-Mouse CD41 phycoerythrin-cyanine7 (eBioscience: 25-0411-82) antibodies for 15 min. Labelled cells were washed with MACS separation buffer and run through a Cyan ADP flow cytometer and were analysed using Summit 4.3 or FlowJo programs. The marker profiles for different cell population are as follows: HE (KIT+, Tie2+ and CD41−), HE2 (KIT +, Tie2+ and CD41+) and CD41 progenitors (KIT+, Tie2− and CD41+).

### Western blotting

For western blots, protein samples were run on a 4–20% gradient polyacrylamide gel (Biorad 456-8093) and transferred to a nitrocellulose membrane. The membrane was blocked in 5% milk in Tris Buffered Saline (TBS) for 1 h at room temperature. The blots were hybridized with primary antibody overnight at 4 °C and washed with TBS Tween (TBST), four times, 5 min each. The blots were then incubated with an appropriate horseradish peroxidase-conjugated secondary antibody (1:20,000) for 1 h at room temperature followed by four washes with TBST 5 min each. The blot was developed using the chemiluminescent reagent (SuperSignal West Pico Chemiluminescent substrate, Thermo scientific 34080). The primary antibodies and dilutions used in this study were anti-AML1 (Cell Signalling Technology #4334, 1:1,000), anti-Runx1/AML1 (Abcam ab23980, 1:3,000) and anti-GAPDH (Cell Signaling Technology 14C10, 1:20,000).

Uncropped images of all blots can be found in the Supplementary Information (Supplementary Fig. 8)

### Progenitor expansion

Floating progenitors from day 2 or day 3 blast culture were seeded in progenitor expansion media at a density of 1 × 10^6^ cells per ml. The progenitor expansion medium was prepared by supplementing IMDM with 10% horse serum, 100 units per ml each penicillin, streptomycin, 1 mM glutamine, stem cell factor (20 ng ml^−1^), Flt3 ligand (10 ng ml^−1^) and thrombopoietin (25 ng ml^−1^).

### Replating assay

For the replating assay, the cells were cultured in methylcellulose MethoCult (StemCell technologies M3134) supplemented with interleukin-3, interleukin-6, granulocyte–macrophage colony-stimulating factor (all 10 ng ml^−1^) and stem cell factor (100 ng ml^−1^). Cells (0.5–1 × 10^4^) were seeded per plate and incubated for 7–8 days. The cells were harvested by adding PBS to the plates and replated. The EBs were harvested by centrifugation and dissociated with trypsin. Cells were then washed in PBS and 10,000 were plated in triplicate.

### mRNA expression analysis

Complementary DNA was prepared from the mRNAs using MMLV-RT (Promega M170A) as per the manufacturers’ recommendations. Real-time PCR was performed with SYBR Green PCR master mix (Life technologies, 4309155) in an ABI Stepone qPCR or 7900HT machine. PCR primers can be found in Supplementary Table 1.

### Microarray and data analysis

For RNA extraction, the cell pellet was resuspended in 800 μl Trizol and stored at −80 °C until used. Before isolation, Trizol was thawed and 200 μl chloroform was added to the Trizol. The cells were vortexed and pelleted. The aqueous phase was collected and an equal volume of isopropanol was added and mixed. The mixture was loaded onto a RNeasy Minelute column (Qiagen, 74204) and purified as per the manufacturer’s instructions. RNA concentration was determined by a nanodrop and its integrity checked using an Agilent Bioanalyzer. The microarrays used were Agilent SurePrint G3 Mouse 8X60K microarrays (catalogue number: G4852A-028005).

### Chromatin immunoprecipitation

For ChIP, the cells were fixed with 1% formaldehyde (Thermo Scientific, 28906) for 10 min at room temperature. Then it was quenched with 1/10th volume of 2 M glycine for 5 min followed by two washes with PBS. The PBS was completely removed and the pellet was stored at −80 °C till used. All the solutions used in ChIP (except the wash and elution buffers) contained 1 × Halt protease inhibitor cocktail (Thermo Scientific 87786). Before ChIP, the cells were thawed on ice and resuspended in ice-cold Buffer A (10 mM HEPES, pH 8.0, 1 mM EDTA pH 8.0, 0.5 mM EGTA, pH 8.0, 0.25% TritonX-100). The cells were shaken for 10 min at room temperature and pelleted at 500 g for 10 min. The supernatant was decanted, pellet was resuspended in ice-cold buffer B (10 mM HEPES, pH 8.0, 200 mM NaCl, 1 mM EDTA pH 8.0, 0.5 mM EGTA, pH 8.0, 0.01% TritonX-100) and incubated at room temperature for 10 min. After pelleting, the cells were resuspended in ChIP buffer 1 (150 μl for 2 million cells, 25 mM Tris pH 8.0, 150 mM NaCl, 2 mM EDTA pH 8.0, 1% TritonX-100 and 0.25% SDS), and sonicated for 20–25 cycles (30 s on and 30 s off). Two volumes of ice-cold ChIP dilution buffer II (25 mM Tris pH 8.0, 150 mM NaCl, 2 mM EDTA pH 8.0, 1% TritonX-100, 7.5% glycerol) was added to the tube and centrifuged for 10 min. The supernatant was collected without disturbing the pellet. About 5% of the chromatin was stored in a separate tube for input. Antibody-coated Dynabeads (Invitrogen) were prepared as per the manufacturer’s instructions by incubating the beads overnight with the antibody. Before mixing with the sonicated chromatin, the antibody was removed and the beads were washed twice with PBS. The sonicated chromatin was added to the beads and rotated at 25 r.p.m. at 4 °C for 2–3 h. After incubation, the beads were separated from the chromatin suspension by keeping the tubes on a magnetic stand and the supernatant was removed. The beads were washed once with ice-cold low salt buffer (20 mM Tris pH 8.0, 150 mM NaCl, 2 mM EDTA pH 8.0, 1% TritonX-100, 0.1% SDS), twice with high salt buffer (20 mM Tris pH 8.0, 500 mM NaCl, 2 mM EDTA pH 8.0, 1% TritonX-100, 0.1% SDS), twice with LiCl buffer (10 mM Tris pH 8.0, 250 mM lithium chloride, 1 mM EDTA pH 8.0, 0.5% NP40, 0.5% sodium-deoxycholate) and once with TE-NaCl buffer (TE pH 8.0 containing 50 mM sodium chloride). All the washes were done by rotating the tubes for 5-10 min at room temperature with cold wash solutions. The chromatin was eluted twice from the beads using 2 × 50 μl elution buffer (100 mM NaHCO_3_, 1% SDS) and shaking at 1,500 r.p.m. for 15 min at room temperature. After adding 4 μl 5 M sodium chloride and proteinase K, the pooled eluate was incubated at 65 °C overnight to reverse the crosslinks. The DNA was recovered by adding 1.8 volumes of Ampure beads (Beckman Coulter A63881). The beads were washed twice with 80% ethanol and the chromatin immunoprecipitated DNA was eluted from the Ampure beads in 0.1 × TE. ChIP quantitative PCR primer sequences can be found in Supplementary Table 2.

### Library preparation

Library preparation was performed using the TruSeq ChIP sample prep kit (Illumina 15034288), with minor modifications. The size of the DNA fragments excised from gel after PCR, varied from 200 to 700 bp. Libraries were validated by doing quantitative PCR for positive and negative control regions and libraries with no or very low signals from negative controls were chosen for sequencing. The DNA quality assessed by running 1 μl of the library on an Agilent Technologies 2,100 Bioanalyser and the library concentration was determined by library quantification kit (Kapa Biosystems, Illumina KK4835). The libraries were pooled and subjected to massively parallel DNA sequencing on an Illumina Genome Analyzer.

### Data analysis

The microarray gene expression scanned images were analysed with Feature Extraction Software 10.7.1.1 (Agilent; protocol GE1_107_Sep09, Grid: 028005_D_F_20100614 and platform Agilent SurePrint G3 Mouse GE 8x60K). The raw data output by Feature Extraction Software was analysed using the LIMMA R package^38^ with quantile normalization and background correction using the normexp method^39^ and an offset value of 16. Contrast matrix, lmFit and eBays function were used and a *P* value cut-off ≤0.001 was applied. Only genes with a minimum log2 intensity value equal to or >6.5 in at least one array were selected as expressed genes. Genes that changed expression at least twofold up or down with respect to ±Dox or genes that changed expression at least twofold up or down through differentiation from HE cells progressing to progenitor cells were considered as differentially expressed.

The principal component analysis was carried out on the RNA level values of the probe set intensity within each experiment and was calculated using ‘prcomp’ function implemented in R (R Core Team, 2013) and the scatterplot3d R package was used for the principal component analysis three-dimensions plots.

Pearson’s correlation coefficients were calculated between samples using log2 of the gene signal intensity. A correlation matrix was generated and Pearson correlation coefficients are displayed after hierarchical clustering as a heat map (Fig. 4b).

Clustering of gene expression was carried out on signal intensity and on fold changes for all differentially expressed genes associated with at least a twofold change. Hierarchical clustering was used with Euclidean distance and average linkage clustering. Heat maps were generated using Mev from TM4 microarray software suite^40^. We then grouped gene expression fold changes according to patterns of expression throughout differentiation (Supplementary Fig. 3a). We identified 27 groups of expression patterns, the codes of 12 changing patterns that hold more than a minimum of 30 genes were displayed as a heat map (Supplementary Fig. 3a), where 1 denotes upregulated, 0 is downregulated and 2 for genes that are invariant and whose expression was unchanged. Fold changes of each of the 12 patterns individually were box plotted. (Supplementary Fig. 3a; right panel)

Gene ontology analysis was performed using BiNGO^41^ and ClueGO tools^42^ using Hypergeometric for overrepresentation and Benjamini and Hochberg (false-discovery rate) correction for multiple testing corrections. KEGG pathway network analysis was performed using ClueGO tools^42^ with kappa score=0.3. Functionally grouped KEGG pathway term networks using kappa statistics implemented by ClueGO to link the terms in the network. The right-sided enrichment (depletion) test based on the hypergeometric distribution is used for terms and groups. The groups are created by iterative merging of initially defined groups based on the kappa score threshold. The relationship between the selected terms is defined based on their shared genes and the final groups are randomly coloured where functional groups represented by their most significant term. One, two or more colours represents that a gene/term is a member of one, two or more groups, respectively. The size of the nodes reflects the enrichment significance of the terms. The network is automatically laid out using the layout algorithm supported by Cytoscape.

The GSEA software^43^ was used to perform GSEA on group of genes against expression data taken from our previously published data. The microarray gene expression data of human Kasumi-1 cells analysis were described in ref. 6. The microarray gene expression data for iRUNX1 system were described in ref. 17. The *P* value and the false-discovery rate q-value are displayed on the enrichment plot.

### Analysis of ChIP-seq data

The sequence reads in fastq format were mapped to the mm10 mouse genome build using BWA^44^ The resulting alignment files were used to generate density maps using bedtools^45^ and data was displayed using the UCSC Genome Browser.

Regions of enrichment (peaks) of ChIP data were identified using MACS 1.4 (ref. 46) and cisGenome^47^ software. The resulting peaks common for the two peak calling methods were considered for further analysis. Peak overlaps, gene annotations were performed using in-house scripts. Peaks were allocated to genes if located in either their promoters or within the region of 500 bp downstream and 2,000 bp upstream of the transcription start sites, as intragenic if not in the promoter but within the gene body region, or if intergenic, to the nearest gene located within 100 kb.

Hierarchical clustering with Euclidean distance and complete linkage clustering was used for clustering of TFs (Fig. 5c) based on similar binding patterns of different ChIP-seq data, in HE, progenitors and cultured progenitors cells. The peaks for all transcriptional factors were intersected and merged when overlapping. The read counts for all union peaks were normalized by total number of reads and then Pearson’s correlation coefficients were calculated between samples using the normalized read counts. A correlation matrix was generated and Pearson correlation coefficients are displayed after hierarchical clustering as a heat map. Colours in the heat map (Fig. 5c) indicate the similarity of association between each pair of TFs.

Analysis of RUNX1 profiles (Fig.7c) was performed as follows: Common RUNX1 and RUNX1-ETO peaks or RUNX1-specific peaks were used as reference coordinates against all aligned reads for RUNX1 before and after RUNX1/ETO induction. Mean read density profiles were calculated for each 50-bp-sized bin around peak summits up to ±5000, bp, these were normalized by the total RUNX1 read counts.

Overlaps between ChIP-seq peaks were defined by requiring the summits of two peaks to lie within ±200 bp. The *P* value for calculating the significance of peak overlaps between peaks was obtained by bootstrapping (50,000 iterations). A random peak set (24,437 peaks) was obtained from the union of the HE, progenitors and cultured progenitors peaks. For bootstrapping, peak sets of 400-b.p.s. width and a population equal to the actual peak populations were randomly obtained from this random set. The mean and the s.d. for the total overlap between one actual peak set and a two random peak set (iteratively exchange between the three peak sets) were calculated and compared with the actual three sets overlap, to obtain the z scores. The *P* value was calculated from the z score using ‘pnorm’ function in R. HE, progenitors and cultured progenitors peaks overlap were found to be significant, with z scores of 23.36 (*P* value <5.45e–121; Fig. 5b). The *P* value for calculating the significance of gene overlaps was carried out similarly using bootstrapping where here random gene set (13,841 genes) was obtained from the union of the HE, progenitors and cultured progenitors genes. HE, progenitors and cultured progenitors genes overlap were found to be significant, with *P* value<4.6e–308). (Fig. 5b).

### Motif analysis

*De novo* motif analysis was performed on non-promoter (distal) peaks using HOMER^48^. Motif lengths of 6, 8, 10, 12 and 14 bp were identified within ±200 bp from the peak summit and a random background sequence option was used. The motif matrices generated by HOMER were scanned against JASPAR with the use of STAMP to identify similarity to known TF-binding sites^49^. The top enriched motifs with a significant log *P* value score were recorded. The annotatePeaks function in HOMER was used to find occurrences of motifs in peaks and distribution of motif density around the peak/motif summit. In this case, we used the discovered motif position weight matrices.

The z score for calculating the significance of motif occurrences within RUNX1-specific distal peaks (Supplementary Fig. 7f) was obtained by bootstrapping, a motif positions search was done within ±200 bp from RUNX1-ETO peaks centre. The distance between the centres of each motif pair was calculated and the motif frequency was counted if the first motif is within 20 bp distance from the second motif. For bootstrapping, peak sets of 400 bps width and a population equal to that of RUNX1 distal-specific peaks were randomly obtained from the union of all RUNX1 and RUNX1-ETO distal peaks from both HE and progenitors cells. Motif search was repeated for each random set and then the mean and the s.d. for the total motif frequencies of the random peak sets were calculated and compared with the actual motif frequencies to obtain the z scores. A matrix was generated and z scores were displayed after hierarchical clustering as a heat map. Red colour means that motif pairs are significantly close within 20 bp in the peaks under test.

### Correlation of gene expression and ChIP-seq data

Genes with at least twofold changes in expression (either up or down) that changed expression from −Dox to +Dox or through differentiation changing from HE cells progressing to HE2 were selected and correlated with RUNX1-ETO ChIP-seq bound genes in HE cells. The resulting HE (HE→HE2) correlated genes were then subdivided into eight classes according to patterns of expression, where 1 if increased expression, 0 if decreased expression and 2 if genes are invariant (Fig. 6c), the main six classes are 00: still downregulated, 02: not downregulated, 11: still upregulated, 12: not upregulated, 20: downregulated, 21: upregulated. Gene ontology analysis and KEGG pathways were performed on these six classes of target HE genes. Genes with at least twofold changes in expression (either up or down) that changed expression from −Dox to +Dox from cultured progenitors were correlated with RUNX1-ETO ChIP-seq bound genes in cultured progenitor cells.

## Additional information

**Accession codes**: All gene expression and DNA-sequencing data are deposited under accession number GSE64625 at NCBI.

**How to cite this article**: Regha, K. *et al*, Developmental-stage-dependent transcriptional response to leukaemic oncogene expression. *Nat. Commun*, 6:7203 doi: 10.1038/ncomms8203 (2015).

## Supplementary Material

Supplementary Figures and Tables Supplementary Figures 1-8 and Supplementary Tables 1-2

Supplementary Data 1 Expression values of all expressed genes in HE, HE2, CD41 progenitors or myeloid (late) Progenitors

Supplementary Data 2 List of gene classes for all differentially expressed genes after RUNX1/ETO induction in HE, HE2 and Progenitor cells as well as their associated GO terms (related to Figure 3a).

Supplementary Data 3 Sheets 1-13: list of genes bound by RUNX1-ETO in HE1 that are differentially expressed through differentiation from HE1 to HE2 and their associated GO and KEGG pathway terms Sheets 14-18: list of genes bound by RUNX1-ETO in HE1 and cultured progenitors which are differentially expressed after RUNX1/ETO induction and their associated GO terms (related to Figure 6)
