# Supplementary material for: Developmental-stage-dependent transcriptional response to leukaemic oncogene expression
Source: Nat Commun. 2015 May 28;6:7203. doi: 10.1038/ncomms8203 (PMC4458875; doi:10.1038/ncomms8203)
Supplement: Supplementary Figures and Tables — Supplementary Figures 1-8 and Supplementary Tables 1-2 [file ncomms8203-s1.pdf]

Supplementary Figure 1

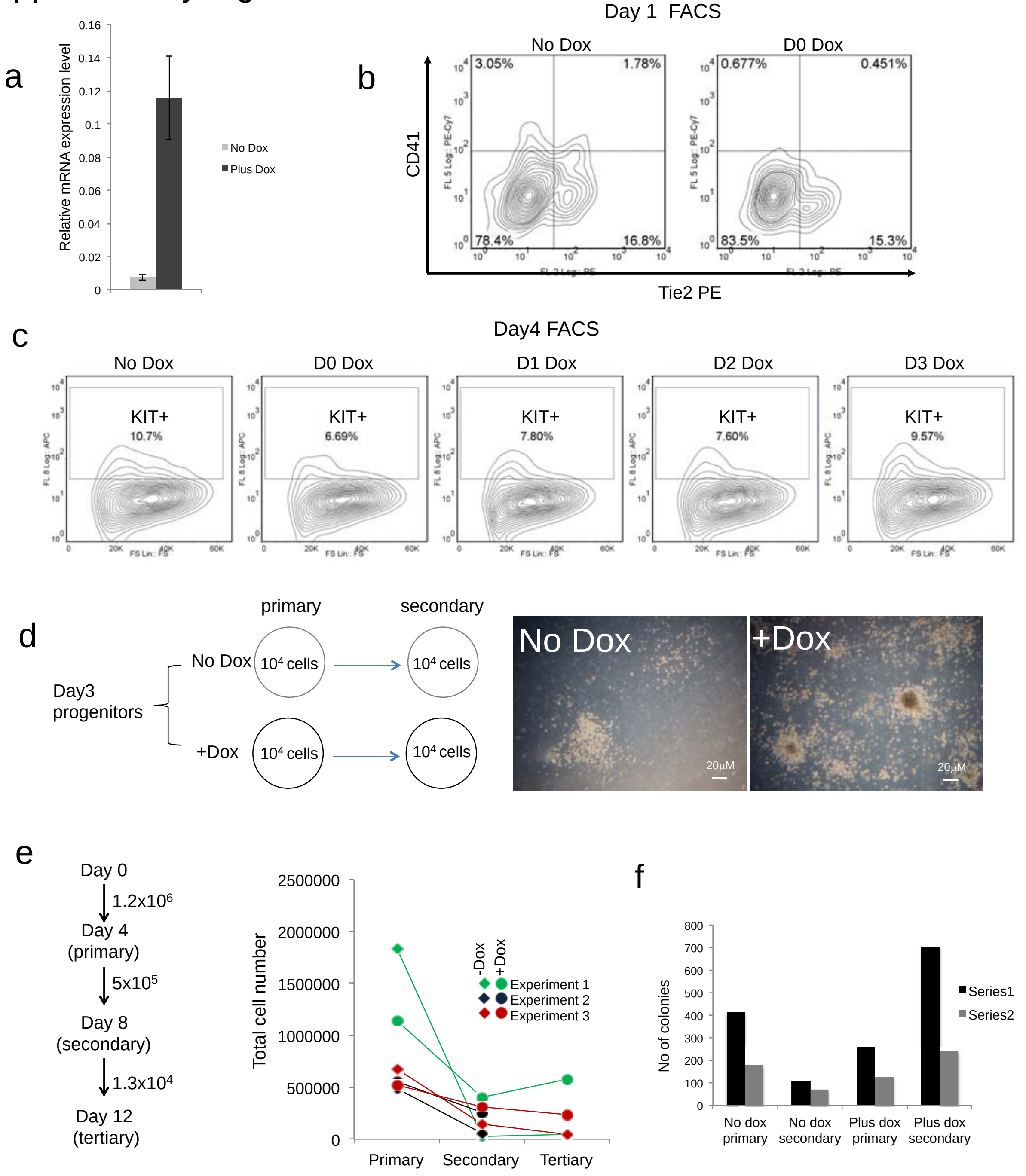

Supplementary Figure 1 (related to Figure 1)

(a) Quantitative RT-PCR analysis of RUNX1-ETO cDNA in CD41+ KIT+Tie2- progenitors from un-induced progenitor expansion culture or after overnight Dox induction. Error bars represent standard deviation of 3 biological replicates. (b) On day 1 the blast culture is mostly in the HE stage of differentiation. Flow cytometry profiles of day 1 blast culture stained with Tie2, CD41 and KIT antibodies in un-induced control cultures (left panel) or induced with 0.1 µg/ml doxycycline on day 0 (right panel). (c) KIT expression levels are not affected by RUNX1-ETO induction at any stage. FACS profiles of blast cultures with and without RUNX1-ETO induction at the indicated days stained with Tie2, CD41 and KIT antibodies gated on the KIT positive population. (d) Outline of CFU assay of

CD41+KIT+Tie2- progenitors from day 3 blast culture in methylcellulose with or without doxycycline shown in (e) and photos of representative colony assay plates (right) with the bar representing 20µM. (e) RUNX1-ETO induced cells keep growing longer. Cell count of day 3 CD41+KIT+Tie2- progenitor cultures in progenitor expansion medium. Cell growth was measured 3 times for experiments 1 and 3 and twice for experiment 2 as indicated. (f) Results of a CFU assay of 10,000 KIT+CD41+Tie2- progenitors from day 3 blast culture in methylcellulose with or without doxycycline and re-plated. Colonies were scored 8 days after each plating. The graph shows two independent experiments (series 1 and 2).

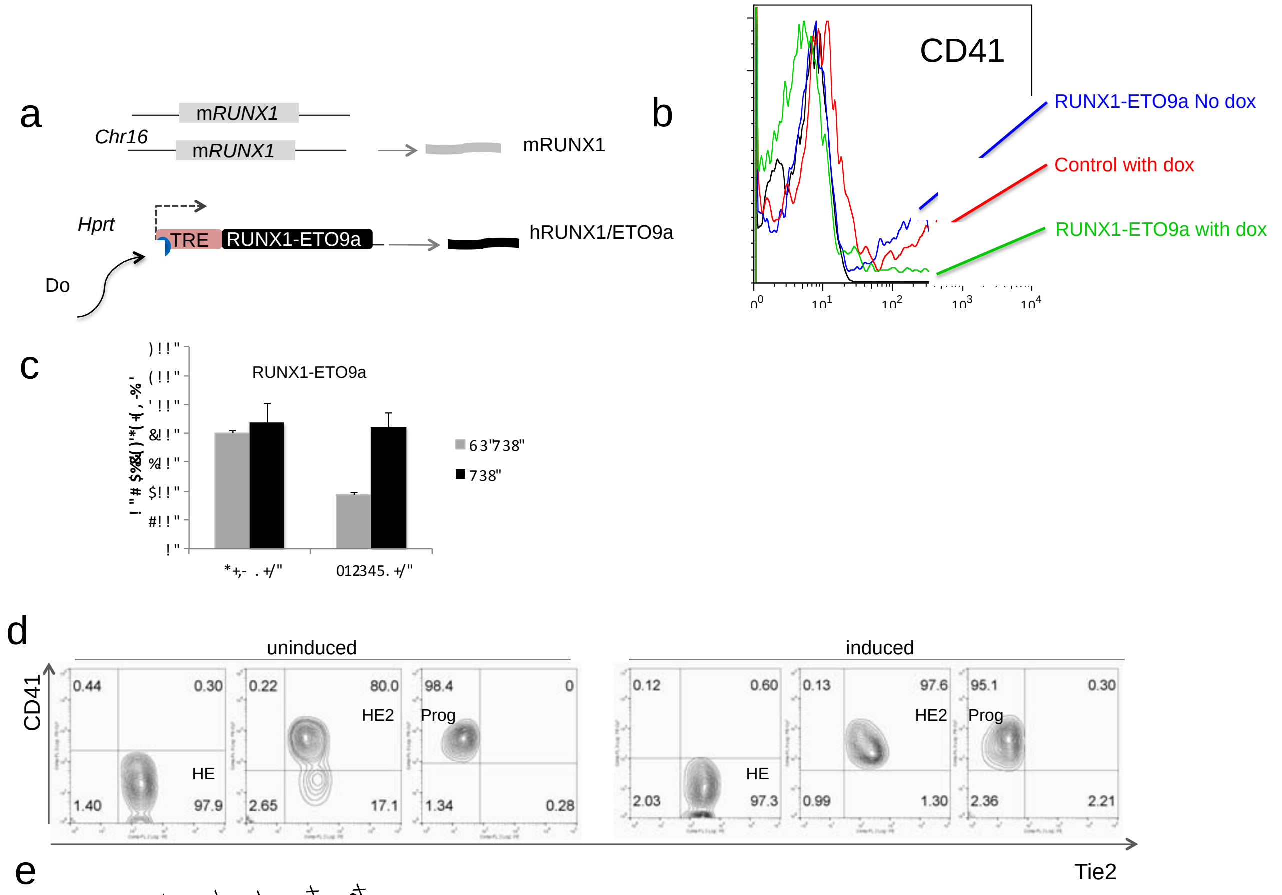

e

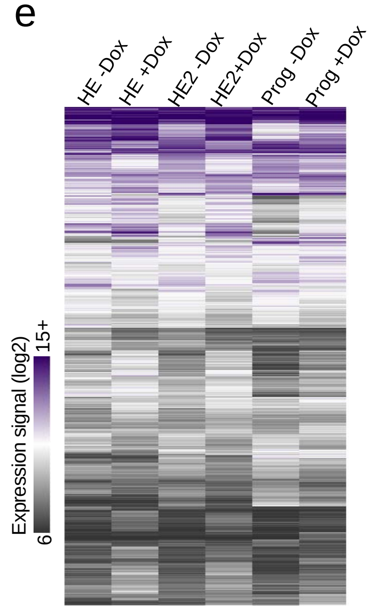

f

		up regulated	down regulated
after RE induction	HE	827	529
	HE2	873	581
	Prog	779	353
	HE→HE2 - Dox	432	605
through differentiation	HE→HE2 +Dox	290	430
	HE→Prog - Dox	439	697
	HE→Prog +Dox	736	950

Supplementary Figure 2 (related to Figure 2)

(a) Schematic representation of the RUNX1-ETO9a inducible ES cell line. (b) FACS evaluation of emergence of CD41 after 2 days of blast culture indicating the defect in emergence of CD41+ cells upon RUNX1-ETO expression. Doxycycline was added at day 0 of blast culture of RUNX1-ETO9a inducible and control ES cell lines. (c) Cells derived from day 5 EBs expressing RUNX1-ETO9a were serially replated in CFU assays in presence or not of Dox. Average numbers (and standard errors) of definitive

haematopoietic colonies generated by 10^4 cells replated in triplicates are depicted. (d) Post sort flow cytometry analysis of purified HE, HE2 and progenitors with and without induction of the full length RUNX1-ETO used for microarray expression studies. (e) Hierarchical clustering of RNA levels of differentially expressed genes through different stages of differentiation from HE to HE2 and HE2 to progenitors before and after day 1 Dox induction. (f) Summary of the number of significantly up- and down-regulated genes in HE, HE2 and CD41+ progenitors after RUNX1-ETO induction and through each stage of differentiation.

Supplementary Figure 3

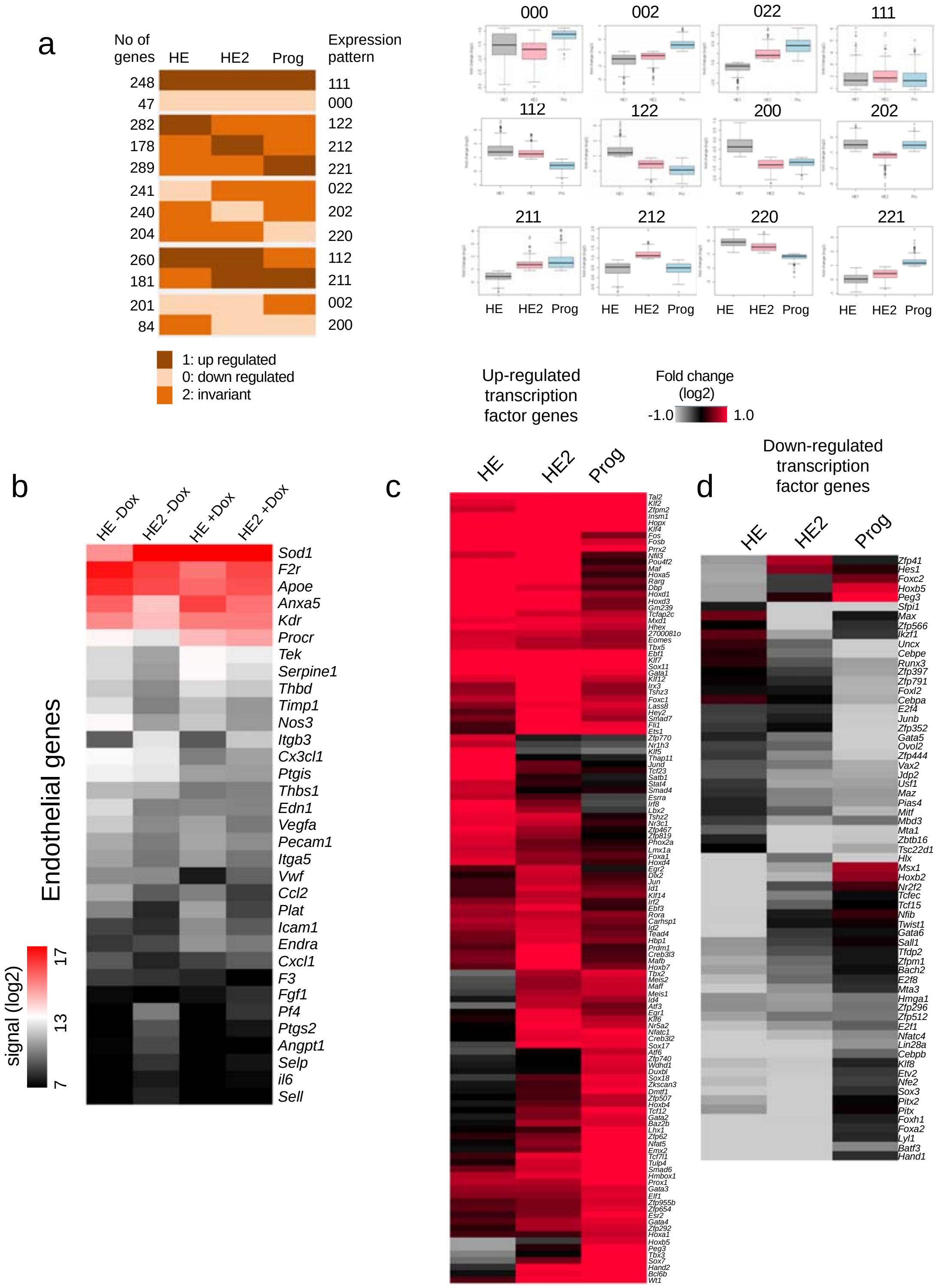

Supplementary Figure 3 (related to Figure 3) (a) Left panel: Genes that show a fold change of 2 or more in expression following RUNX1-ETO induction during differentiation were grouped into different classes. Up- and down-regulated genes could be separated into 12 major clusters according to changes in expression levels during differentiation.

Right panel: Boxplots indicating that the fold changes in gene expression in the 12 major clusters in the left panel are significant. The whiskers were extended down to the minimum value and up to the maximum value. (b) Heatmap representing the absolute endothelial gene RNA levels between RUNX1-ETO induced and un-induced cultures in the HE and HE2. Each line represents the RNA signal of individual genes in the induced and un-induced state. (c) Heat map showing fold change in the expression of transcription factor genes up-regulated in the RUNX1-ETO induced HE, HE2 and progenitors, (d) as in (c), but down-regulated transcription factor genes.

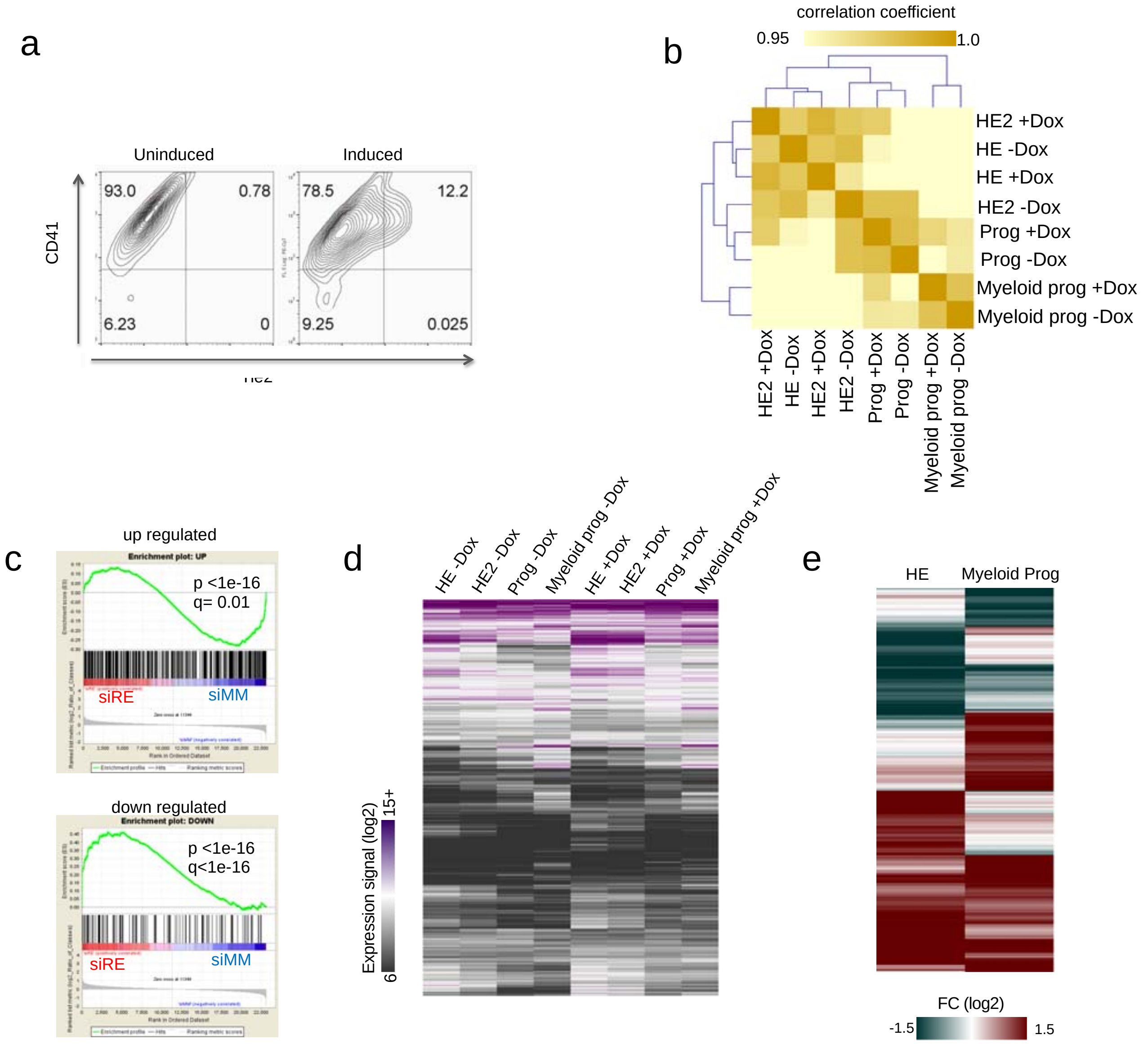

Supplementary Figure 4 (related to Fig. 4) (a) FACS analysis of myeloid progenitors with and without 12 hours of RUNX1-ETO induction analysing CD41 and Tie2 expression. (b) Pearson correlation of gene expression patterns from the different cell types demonstrating how RUNX1-ETO blocks the transition to HE2 and progenitor patterns. (c) GSEA analysis showing a comparison of changes in gene expression caused by RUNX1-ETO between mouse ES-cell derived progenitor cells and human t(8;21) cells with (siRE) and without (siMM) RUNX1-ETO knock-down showing an inverse correlation whereby genes that were up-regulated in the presence of RUNX1-ETO are down-regulated in induced mouse progenitor cells and vice-versa. The p-value and the FDR q-value are displayed on the enrichment plot (d) Heatmap showing the comparison of the gene expression patterns between the different indicated cell populations. (e) Heatmap showing hierarchical clustering of fold changes (FC) in gene expression demonstrating the differential response of HE and myeloid progenitors to RUNX1-ETO induction.

Supplementary Figure 5

a

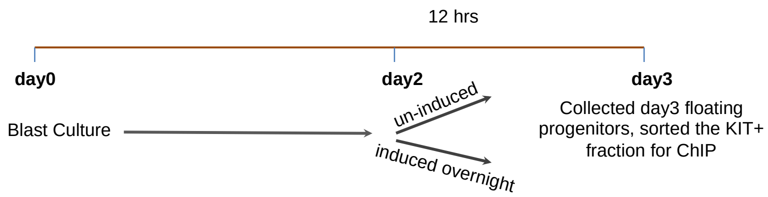

b

Surface marker profile of KIT+ floating progenitors

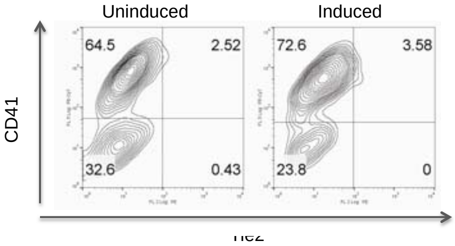

c

Number of aligned reads and peaks

**Dataset**	Number of ChIP peaks	Number of associated genes
RUNX1/ETO HE induced	13,427	7,387
RUNX1/ETO KIT+ progenitors induced	16,852	8,619
RUNX1/ETO myeloid progenitors induced	11,261	7,871
RUNX1 myeloid progenitors un-induced	11,518	7,800
RUNX1 myeloid progenitors induced	9,449	6,823
RUNX1/ETO HE un-induced	181	110

d

1. Enriched motifs in shared peaks between all populations

2. Enriched motifs in peaks specifically shared between HE and KIT+ floating progenitors

**Motif**	**Match**	**Score (-log p)**	**% target**	**Motif**	**Match**	**Score (-log p)**	**% target**
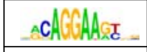	**ETS**	**565.5**	**45.7**	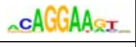	**ETS**	**563.0**	**49.2**
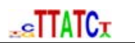	**GATA**	**485.2**	**35.3**	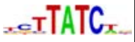	**GATA**	**287.0**	**23.1**
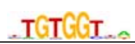	**RUNX**	**286.6**	**42.5**	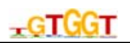	**RUNX**	**222.2**	**27.2**
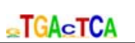	**AP1**	**63.6**	**16.3**	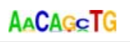	**E-box**	**53.5**	**22.0**
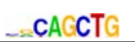	**E-box**	**39.5**	**25.2**	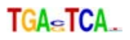	**AP1**	**36.9**	**11.1**

Supplementary Figure 5 (related to Figure 5). (a) Outline of the experimental scheme for the induction and collection of KIT+ floating (non-adherent) progenitors from day 3 blast culture representing cells that had undergone the EHT. (b) FACS analysis of isolated KIT+ progenitors with and without 12 hours of RUNX1-ETO induction demonstrating that the majority of such cells also expresses the CD41 marker. (c) Number of peaks and genes obtained in the different ChIP-seq experiments. (d) Enriched sequence motifs in the peaks shared between all populations (left) and in the peaks specifically shared between HE and KIT+ progenitors.

Supplementary Figure 6

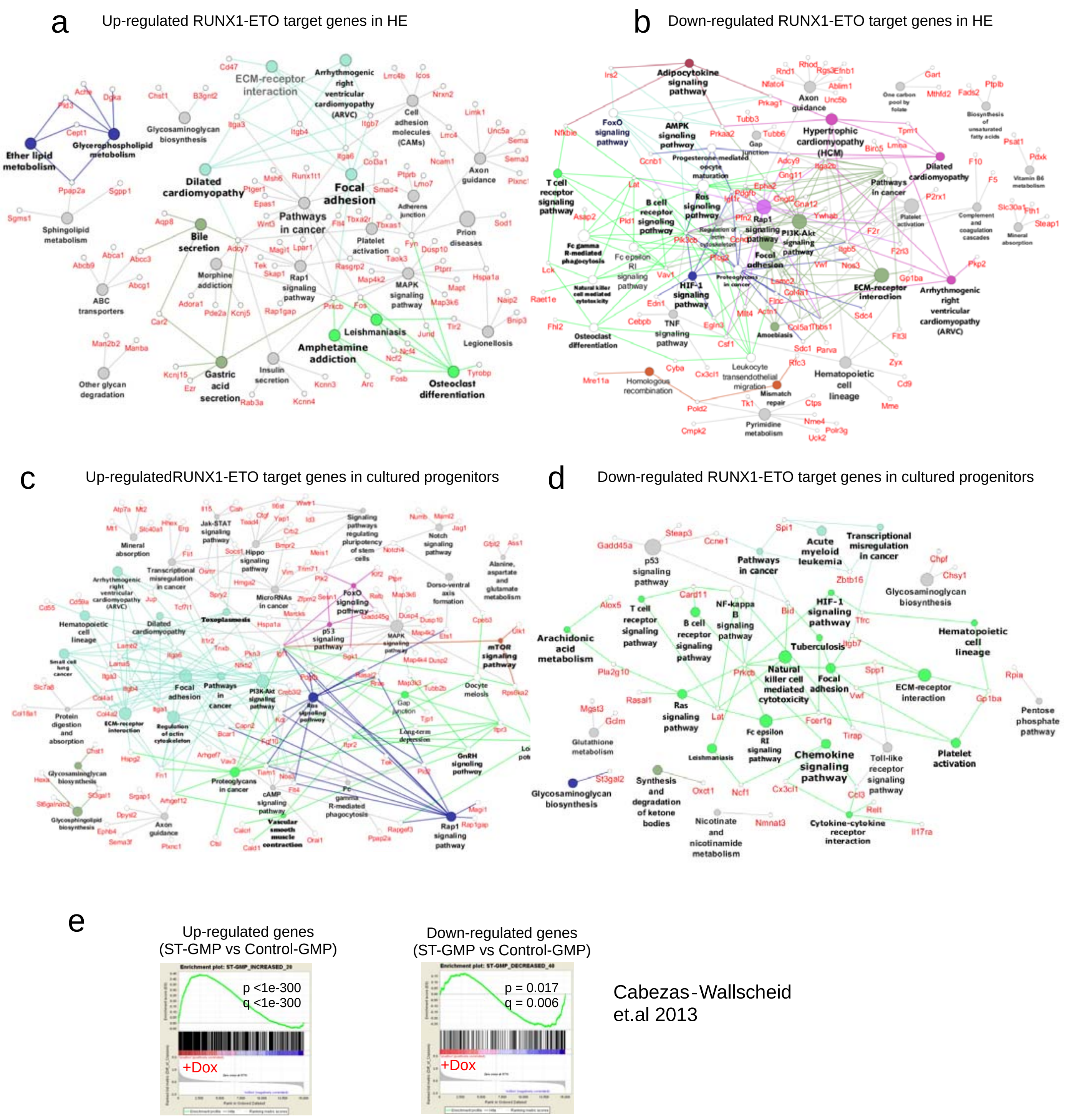

Supplementary figure 6 (related to Figure 6). (a – d) KEGG pathway analysis of RUNX1-ETO target genes. Functionally grouped KEGG pathway terms in the network are used to link the terms in the network using kappa statistics implemented by ClueGO. Functional groups are represented by their most significant term. One, two or more colours represents a gene/term being a member of one, two or more groups respectively. The size of the nodes reflects the enrichment significance of the terms. (a) Genes and

pathways up-regulated in the HE, (b) genes and pathways down-regulated in the HE, (c) genes and pathways up-regulated in myeloid progenitors (d), genes and pathways down-regulated in myeloid progenitors. (e) GSEA demonstrating a strong correlation between the patterns of RUNX1-ETO responsive genes from the ES cell derived myeloid precursor cells described in this study and the fold change obtained with RUNX1-ETO expressing granulocyte-macrophage progenitors (GMPs) compared to control cells.

Supplementary Figure 7

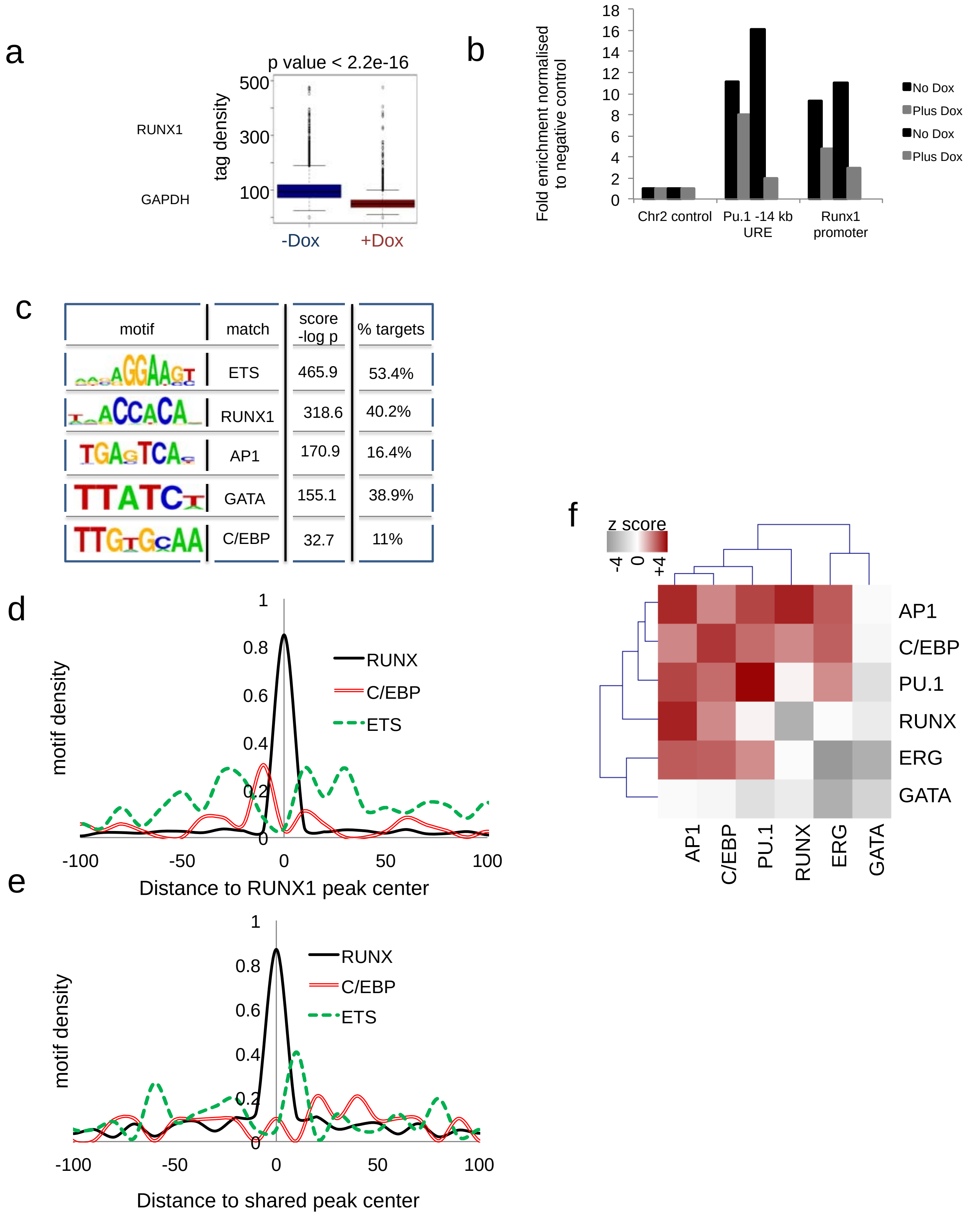

Supplementary figure 7 (related to Figure 7) (a) Box-plots demonstrating that the changes in RUNX1 occupancy at RUNX1-ETO binding sites after RUNX1-ETO induction are significant. The t-test was used to calculate the p value. The whiskers were extended down to the minimum value and up to the maximum value. (b) Manual validation of ChIP-

Seq results showing a reduction of RUNX1 binding at the indicated target sites by qPCR. The experiment was performed twice and the respective values are shown. (c) Transcription factor motifs overrepresented in the RUNX1-only distal peaks. (d) RUNX1, C/EBP and ETS binding motif density within RUNX1-only peaks containing RUNX1 motifs (675), C/EBP (214) as well as ETS (1025) motifs. (e) Motif density of RUNX1 (631), ETS (956) and C/EBP (124) motifs in shared sites bound by RUNX1-ETO and RUNX1. (f) Analysis of pairwise motif clustering (bootstrapping analysis) from RUNX1-only peaks with RUNX1 motifs demonstrating that the co-localization of RUNX1, C/EBP and AP-1 motifs is statistically significant. The distance was calculated within 20bp and compared to random sets. The red colour means the two motif pairs are significantly closer to each other than randomly expected. The random sets were extracted from the union of all RUNX1 and RUNX1-ETO peaks in cultured progenitors and HE.

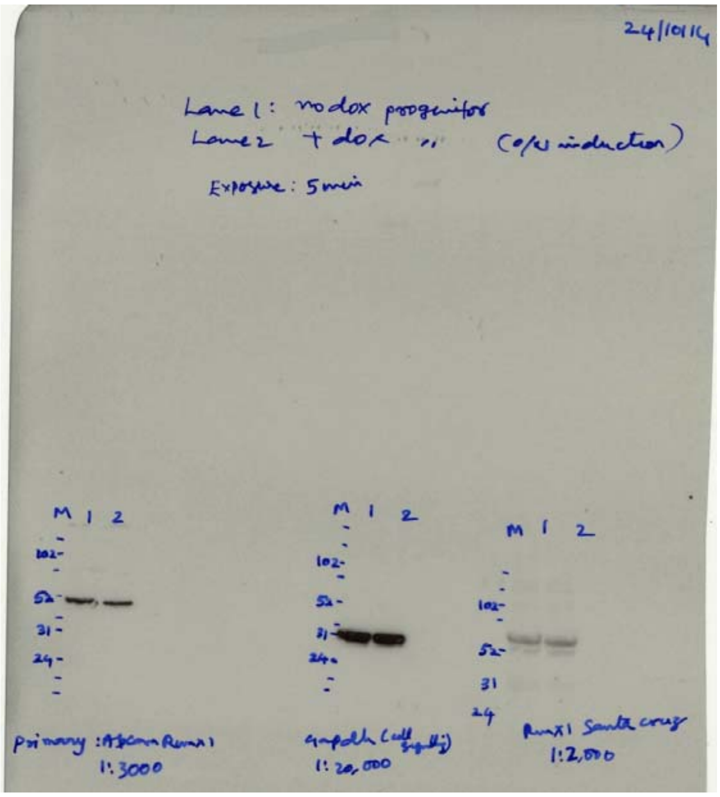

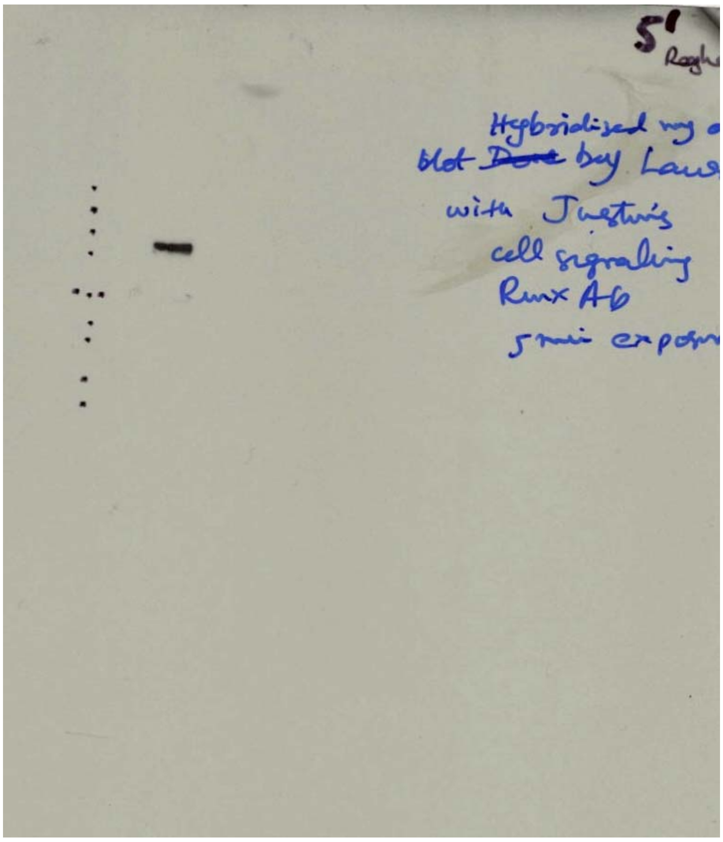

Supplementary Figure 8: Original Blots for the generation of Figure 1b

SUPPLEMENTARY TABLES:

Supplementary Table 1: qPCR primers for expression analysis

	Forward	Reverse
Sox17	CTAAGCAAGATGCTAGGCAAG	TACTTGTAGTTGGGGTGGTCC
Runx1	AGCGGTAGAGGCAAGAGCTTC	CGGATTTGTAAAGACGGTGATG
Pu.1	CCATAGCGATCACTACTGGGATTT	TGTGAAGTGGTTCTCAGGGAAGT
Tie2/Tek	TGCAACTGAAGAGAGCAAATG	TCAAGCACAGGATAAATTGTG
RUNX1-ETO	TCAAAATCACAGTGGATGGGC	CAGCCTAGATTGCGTCTTCACA
Runt Domain	AACAAGACCCTGCCCATCGCTTTC	CATCACAGTGACCAGAGTGCCAT

Supplementary Table 2: ChIP-qPCR primers

	Forward	Reverse
Runx1 promoter	CAGCAGGCAGGACGAATCA	CGCCTATGCTGTGGGTTGA
Pu.1 -14 kb	GCCCAGGCTAGGGAAGTTTG	GAGAGCAGAGCACTTCATGGCTA
Chr2	AGGGATGCCCATGCAGTCT	CCTGTCATCAGTCCATTCTCCAT
